# A_2_B_*n*–1_Pb_*n*_I_3*n*+1_ (A = BA, PEA; B = MA; *n* = 1, 2): Engineering Quantum-Well Crystals for High Mass Density and Fast Scintillators

**DOI:** 10.1021/acs.jpcc.3c00824

**Published:** 2023-04-26

**Authors:** Md Abdul Kuddus Sheikh, Dominik Kowal, Muhammad Haris Mahyuddin, Roberto Cala’, Etiennette Auffray, Marcin Eugeniusz Witkowski, Michal Makowski, Winicjusz Drozdowski, Hong Wang, Christophe Dujardin, Daniele Cortecchia, Muhammad Danang Birowosuto

**Affiliations:** † Łukasiewicz Research Network-PORT Polish Center for Technology Development, Stabłowicka 147, Wrocław 54-066, Poland; ‡ Research Group of Advanced Functional Materials and Research Center for Nanoscience and Nanotechnology, Institut Teknologi Bandung, Jl. Ganesha 10, Bandung 40132 Indonesia; § Dipartimento di Fisica, Università di Milano-Bicocca, Milan 20126, Italy; ∥ CERN, Esplanade des Particules 1, 1211 Meyrin, Switzerland; ⊥ Institute of Physics, Faculty of Physics, Astronomy, and Informatics, Nicolaus Copernicus University in Toruń, ul. Grudzia̧dzka 5, 87-100 Toruń, Poland; # School of Electrical and Electronic Engineering, Nanyang Technological University, Singapore 639798, Singapore; % Institut Lumière Matière, UMR5306, Université Claude Bernard Lyon1 and CNRS Lyon, 69622 Lyon, France; & Dipartimento di Chimica Industriale “Toso Montanari”, Università di Bologna, 40136 Bologna, Italy

## Abstract

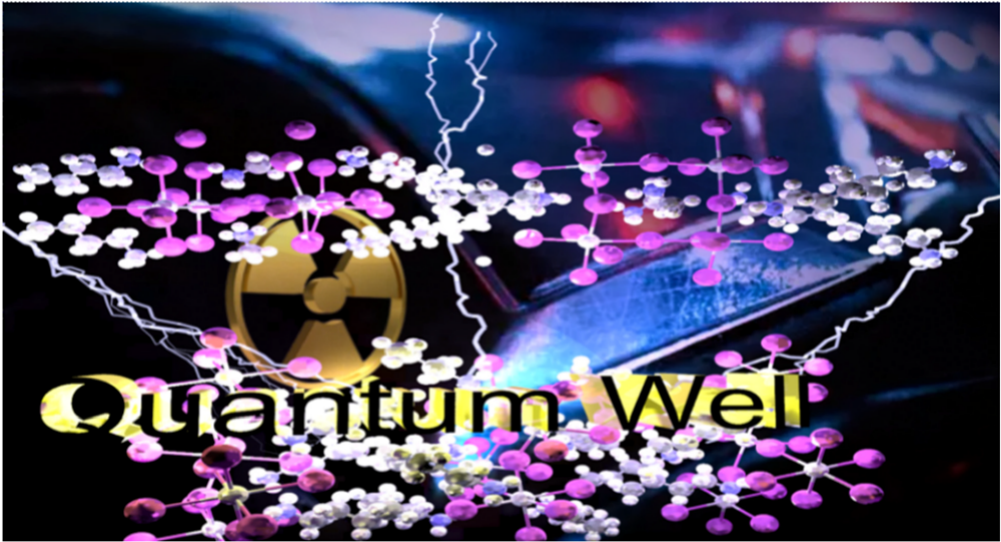

Quantum-well (QW) hybrid organic–inorganic perovskite (HOIP) crystals, e.g., A_2_PbX_4_ (A = BA, PEA; X = Br, I), demonstrated significant potentials as scintillating materials for wide energy radiation detection compared to their individual three-dimensional (3D) counterparts, e.g., BPbX_3_ (B = MA). Inserting 3D into QW structures resulted in new structures, namely A_2_BPb_2_X_7_ perovskite crystals, and they may have promising optical and scintillation properties toward higher mass density and fast timing scintillators. In this article, we investigate the crystal structure as well as optical and scintillation properties of iodide-based QW HOIP crystals, A_2_PbI_4_ and A_2_MAPb_2_I_7_. A_2_PbI_4_ crystals exhibit green and red emission with the fastest PL decay time <1 ns, while A_2_MAPb_2_I_7_ crystals exhibit a high mass density of >3.0 g/cm^3^ and tunable smaller bandgaps <2.1 eV resulting from quantum and dielectric confinement. We observe that A_2_PbI_4_ and PEA_2_MAPb_2_I_7_ show emission under X- and γ-ray excitations. We further observe that some QW HOIP iodide scintillators exhibit shorter radiation absorption lengths (∼3 cm at 511 keV) and faster scintillation decay time components (∼0.5 ns) compared to those of QW HOIP bromide scintillators. Finally, we investigate the light yields of iodide-based QW HOIP crystals at 10 K (∼10 photons/keV), while at room temperature they still show pulse height spectra with light yields between 1 and 2 photons/keV, which is still >5 times lower than those for bromides. The lower light yields can be the drawbacks of iodide-based QW HOIP scintillators, but the promising high mass density and decay time results of our study can provide the right pathway for further improvements toward fast-timing applications.

## Introduction

Hybrid organic–inorganic perovskite (HOIP) crystals have attracted significant attention due to their remarkable properties, including long carrier diffusion length, low defect density, possibly high absorption coefficient, medium exciton binding energy, and tunable bandgap.^1−7^ Such properties make them promising materials for next-generation optoelectronic applications.^8−13^ In addition, the presence of the heavy lead (Pb) element is favorable to the absorption of high-energy X-rays and γ-rays. Despite their widespread applications and below-oven-furnace temperature processability, commercialization of these devices is hindered by their poor environmental stability. Quantum-well (QW) HOIP crystals, A_2_PbX_4_,^14^ have shown remarkable environmental and thermal stability compared to their three-dimensional (3D) counterparts, BPbX_3_ (B = methylammonium (MA)), while preserving significant optical and scintillation properties toward targeted applications.^14−18^ These materials consist of inorganic perovskite slabs intercalated with bulky organic cations that act as spacers between these layers, adopting the crystal structure of the Ruddlesden–Popper (RP) type. In particular, QW HOIP crystals, as direct bandgap materials, have demonstrated high potentialities as scintillating materials for fast timing applications in security, medical diagnosis, industrial sectors, high-energy physics, and materials sciences.^19−22^ They exhibit high light yields >10 photons/keV and short scintillation decay times <15 ns, leading to good coincidence timing resolutions (CTR) < 150 ps.^21,23,10,8^ However, the absorption lengths of typical QW HOIP are >2 times longer than that of the commercial CsI:Tl scintillators,^24^ making the crystals less attractive for high-energy excitation applications, such as time-of-flight positron emission tomography (TOF-PET), which is operating at 511 keV.^20^ In addition, so far QW HOIP scintillators have been reached with bromide of (Br) variants with 3 eV bandgaps (e.g., phenethylammonium lead bromide (PEA)_2_PbBr_4_ and *n*-butylammonium lead bromide (BA)_2_PbBr_4_), still possess scintillation light yields about half from 66 photons/keV of commercial CsI:Tl scintillator.^20^ To improve the yields, one can look to the lower bandgap materials such as 3D HOIP or iodide (I) crystals with 2 eV bandgaps. They may provide higher light yields although so far the light yields were recorded at low temperature.^19^ To achieve better absorption lengths and light yields, one should get higher density materials and smaller bandgaps, respectively.^20^ For the faster decay time, one has to search iodide (I^–^) crystals of QW HOIP, as most crystals exhibit decay times of <2 ns but lower light yield.^16^ This requires a modulation of the crystals properties between those of QW and 3D HOIP structures, which can be synthetically achieved exploiting the versatility of the RP compositions A_2_B_*n*–1_Pb_*n*_I_3*n*+1_ (where *n* is an integer). Such materials allow the full control of the optoelectronic properties either by compositional engineering or by structural modulation exploiting different levels of quantum and dielectric confinement in materials with different dimensionalities (*n*). Among the numerous reported perovskite-based optoelectronic devices, including solar cells,^7,25−27^ field-effect transistors (FETs),^28^ light-emitting diodes (LEDs),^29,30^ and photodetectors,^31^ many are based on powders and thin films. Particularly, PEA_2_MA_*n*–1_Pb_*n*_I_3*n*+1_ compounds, which exhibit multiple quantum-well structures, are extensively investigated for application light-emitting diodes (LEDs) due to their excellent photoluminescence (PL) properties. However, there have been hitherto no reports available on the A_2_B_*n*–1_Pb_*n*_I_3*n*+1_ materials utilized as scintillators.

In this article, we synthesize four different RP iodide-based QW HOIP crystals with *n* = 1, 2 as *n* is the number of 3D structures sandwiched between QW layers. We investigate the crystal structure, optical, and scintillation properties of (PEA)_2_PbI_4_, (BA)_2_PbI_4_, and the corresponding *n* = 2 RP phases (PEA)_2_MAPb_2_I_7_ and (BA)_2_MAPb_2_I_7_. (PEA)_2_PbI_4_ was previously discussed by our group in a short report by comparing different methods in crystal fabrications for light yield optimization.^32^ Here we present the global trend in optical and scintillation properties with other unreported properties from other three iodide structures. We show that (BA)_2_PbI_4_ and (PEA)_2_PbI_4_ crystals exhibit green and red emission with the fastest PL decay time. We further find that *n* = 2 layered perovskite iodide scintillators exhibit a mass density of >3.0 g/cm^3^ and tunable rather small bandgaps <2.1 eV resulting from quantum and dielectric confinement due to the dimensional reduction of the perovskite spacer layers compared to 3D structures. Among these, we observe that only (PEA)_2_MAPb_2_I_7_ shows emission under X- and γ-ray excitations. From all these iodide crystals, we find light yields at room temperature (RT) (1–2 photons/keV) considerably lower than those of (BA)_2_PbBr_4_ and (PEA)_2_PbBr_4_ (10–40 photons/keV), while at 10 K, the light yields are comparable (∼10 photons/keV).^16^ Thus, applications at low temperature are envisaged when a shorter radiation absorption length (∼3 cm at 511 keV) and a faster decay time component (∼0.5 ns) are foreseen. Such results may provide a new pathway for further improvements of these materials toward fast-timing applications.

## Materials and Methods

### Materials

Methylammonium chloride (MACl), 50% aqueous H_3_PO_2_, lead oxide (PbO, 99.999%), phenethylamine (99%), *n*-butylamine (>99%), 57% stabilized hydroiodic acid (HI), lead iodide (PbI_2_, 98%), dimethyl sulfoxide (DMSO, anhydrous), *n*-butylammonium bromide ((BA)Br, ≥98%)), phenethylammonium bromide ((PEA)Br, ≥98%), and lead bromide (PbBr_2_, ≥98%) were purchased from Sigma-Aldrich.

### Synthesis of QW HOIP Crystals

The QW A_2_PbI_4_ crystals were synthesized using a method previously reported by Kowal et al.^32^ The QW A_2_PbBr_4_ crystals were synthesized using a modified version of the previously reported method.^14,33^ A 3 M precursor solution was prepared by dissolving (BA)Br or (PEA)Br and PbBr_2_ in stoichiometric amounts in DMSO under stirring at 100 °C for 2 h. The crystal precipitate was then washed with hexane and dried under vacuum for future characterization. For A_2_MAPb_2_I_7_ crystals, PbO (223.2 mg) and MACl (200 mg for PEA and 33.8 mg for BA) powders were dissolved in aqueous HI solution (2 mL for PEA and 1 mL for BA) with the addition of 50% aqueous H_3_PO_2_ (0.17 mL). Separately, 88.2 μL of PEA and 69.4 μL of BA were neutralized with 0.5 mL of HI 57% w/w, respectively, causing the precipitation of a white solid that redissolved upon heating. The PEA or BA solution was added to the PbO/MACl solution, and the mixture heated at 150 °C under magnetic stirring on a hot plate. The solution was transferred in an oven where it was kept for 24 h, and during this time the temperature decreased from 100 to 20 °C, allowing the growth of red crystals for PEA-based and dark red crystals for BA-based A_2_MAPb_2_I_7_, respectively. These were collected by filtration and dried at 100 °C under vacuum. The obtained perovskite crystals were stored in the glovebox under an inert atmosphere.

### X-ray Diffraction

A Bruker D8 Advance AXS diffractometer was used for measuring powder X-ray diffraction (XRD) spectra of the synthesized compounds.^32^ The device used Cu Kα radiation with 1.5418 Å wavelength. Measurements were conducted at RT, under Bragg–Brentano geometry, 5 s/step scanning velocity, and 0.02° step size. FullProf Suite software was then used to analyze the acquired data.

### PL, TRPL, and Absorption

For PL measurements the samples were excited with the use of picosecond laser diode with repetition rate 30 MHz, 375 and 532 nm peak wavelengths (Master Oscillator Fiber Amplifier, PicoQuant GmbH, Berlin, Germany), pulse duration 50 ps, and 10 mW average power. A microscopic objective with numerical aperture (NA) 0.4 and magnification 20× (Nikon Corporation, Tokyo, Japan) was used for excitation focusing and signal collection. The filtered PL signal was acquired by a high-sensitivity visible light spectrometer (Ocean Optics, Orlando, FL). For TRPL measurements, the repetition rate was reduced to 10 MHz, and the PL signal, selected by bandpass filter 532 ± 25 nm, was coupled to a single-photon avalanche photodiode (APD). The timing response was analyzed by time-correlated single-photon counting electronics (HydraHarp 400, PicoQuant, Germany). A tungsten halogen light source (Ocean Optics LS-1) and same visible light spectrometer as for the PL experiments were used to measure the absorption of the samples in the transmission mode. All measurements were conducted at RT.

### RL, TL, and Afterglow Curves

The X-ray excitation was provided by an Inel XRG3500 X-ray generator Cu-anode tube (45 kV/10 mA). For recording the optical signal, we used an Acton Research Corporation SpectraPro-500i monochromator, a Hamamatsu R928 photomultiplier tube (PMT), and an APD Cryogenic Inc. closed-cycle helium cooler. The crystals were exposed to X-ray radiation for 10 min, and the afterglow curve was recorded at temperature of 10 K. Then, TL glow curves were measured from 10 to 350 K by increasing the temperature, with 0.14 K/s heating rate. Finally, the RL signal was measured from 350 to 10 K by cooling the sample back. The measurement started from the highest temperature as to avoid thermal release of charge carriers which could possibly contribute to the emission yield.

### Pulse Height and Scintillation Decay Measurements

For source of the γ-rays, a ^137^Cs (662 keV, 210 kBq) radioisotope was used, and the converted photons were detected by a PMT (Hamamatsu R878) with 1.25 kV applied voltage. The output was integrated with a charge-sensitive preamplifier (Canberra 2005), and then it fed a spectroscopic amplifier (Canberra 2022) with a shaping time of 2 μs and a TUKAN-8K-USB multichannel analyzer. In the pulse height spectrum, the position of the photopeak was compared with the position of the mean value of the single electron response to obtain the photoelectron yield. The actual light yield for the radiation conversion in photons per MeV was obtained by taking into consideration the spectral matching of the sample luminescence to the PMT characteristics. Scintillation decay measurements were performed by the delayed coincidence single photon counting method.^16^ A ^137^Cs radioactive source, two Hamamatsu photomultiplier tubes (R1104 and R928 for “starts” and “stops”, respectively), a Canberra 2145 time to-amplitude converter, and a TUKAN-8K-USB multichannel analyzer were used.

### DFT Calculations

The Kohn–Sham formulation^34^ as implemented in the Vienna Ab initio Simulation Package (VASP)^35^ was used for the DFT calculations. The projector augmented wave (PAW) method^36^ was used to describe the interaction between ion cores and electrons. The details of the simulation setup and the parameters used were the same as reported in ref (32).

## Results and Discussion

**Figure 1:**
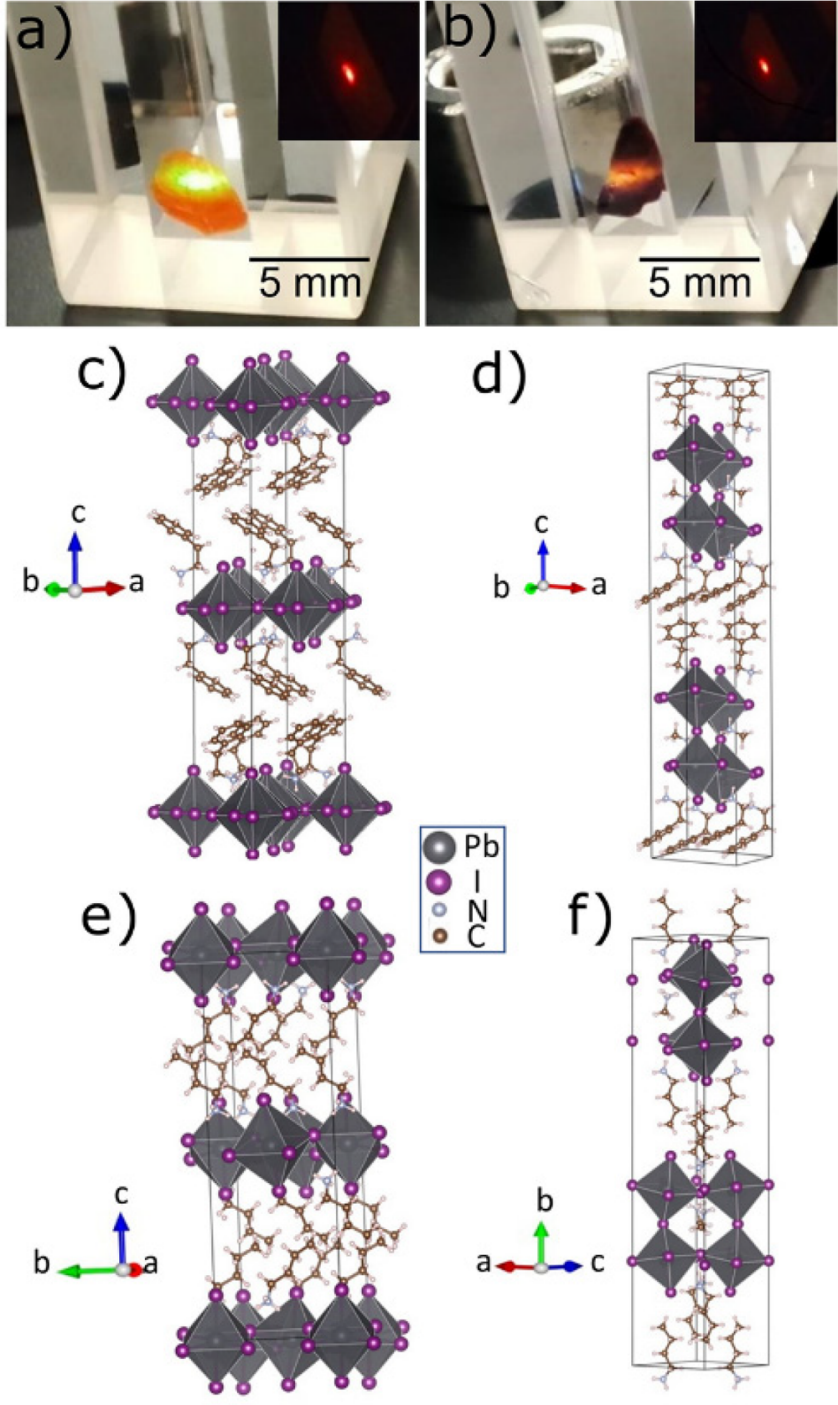
Photographs of (a) (PEA)_2_PbI_4_ and (b) (PEA)_2_MAPb_2_I_7_ crystals under 375 nm laser excitation. The insets correspond to red emission at the 532 nm excitation wavelength. Schematic crystal structures of (c) (PEA)_2_PbI_4_, (d) (PEA)_2_MAPb_2_I_7_, (e) (BA)_2_PbI_4_, and (f) (BA)_2_MAPb_2_I_7_ perovskite.

Photographs of (PEA)_2_PbI_4_ and (PEA)_2_MAPb_2_I_7_ crystals excited by a 375 nm laser beam are displayed in Figures 1a,b, and their crystal structures are shown in Figures 1c,d, respectively. The triclinic (PEA)_2_PbI_4_ and orthorhombic (BA)_2_PbI_4_ belong to the class of two-dimensional (2D) A_2_PbX_4_ (A = PEA, BA; X = Br, I) HOIP and consist of the stack of ⟨100⟩-oriented perovskite inorganic layers, forming a 2D Pb–X octahedra network in alternation with the organic sheets of PEA and BA cations as displayed in Figures 1c,e. Schematically, 2D HOIP crystals exhibit a layered structure,^37,16^ like QW, with inorganic [PbI_6_]^4–^ octahedra sheets separated by another layer of organic ammonium cations. The ball and stick structures of the *n* = 2 compounds A_2_MAPb_2_I_7_ (A = PEA, BA) are shown in Figures 1d,f, where all diagrams include the {PbI_6_} octahedral units. Moreover, PEA^+^ and MA^+^ organic cations were so disordered that the benzene ring and MA^+^ cation could be hardly distinguished. Significant disorder exists in the interlayer cations of (BA)_2_MAPb_2_I_7_ crystal, particularly for the CH_3_CH_2_– tail of butylammonium (the ligand head, NH_3_CH_2_CH_2_– is relatively stable), causing the atoms to move and destabilize the refinement.^37^

**Figure 2:**
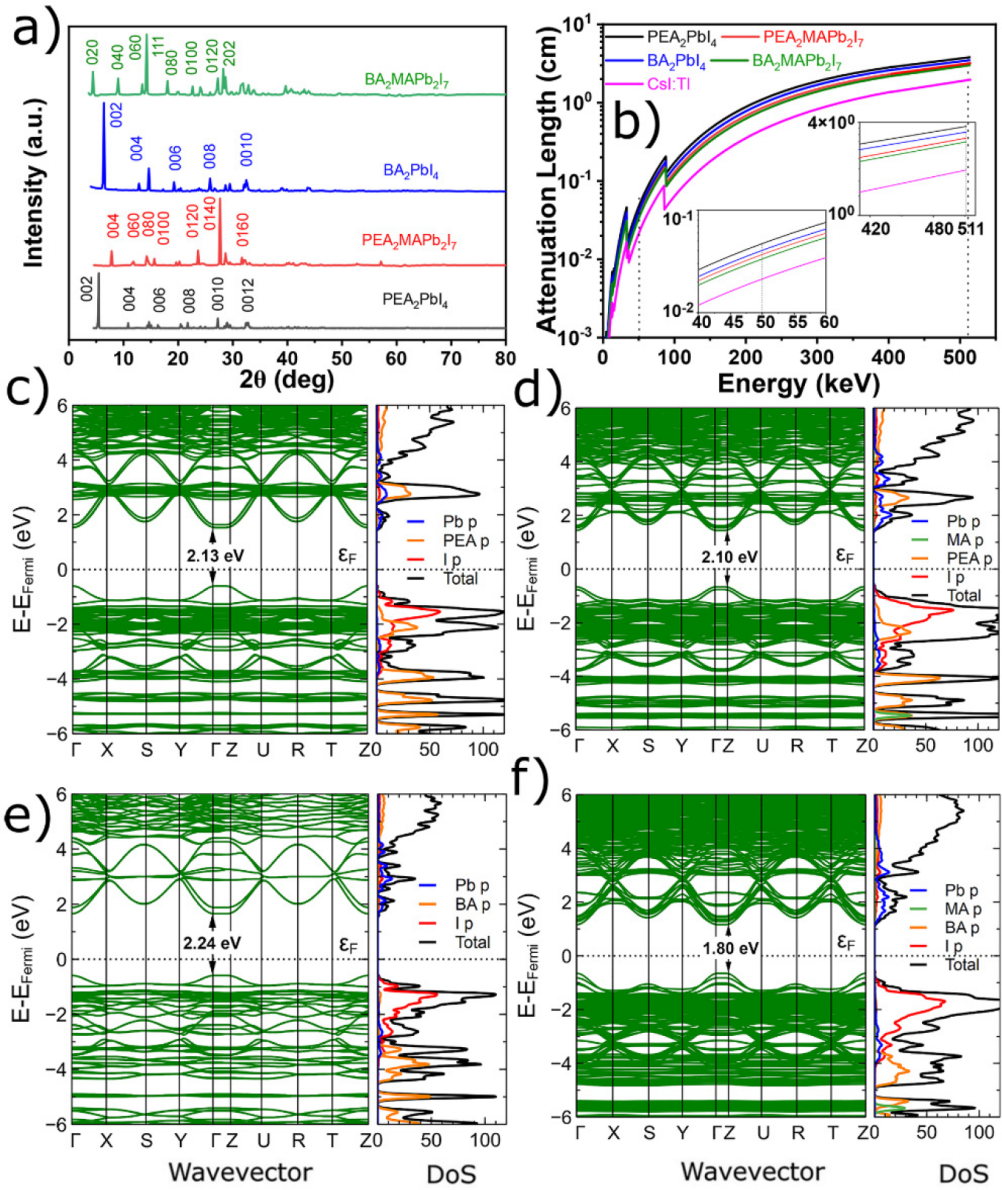
(a) X-ray diffraction pattern of the crystals. (b) Absorption lengths  calculated for X-ray and γ-ray spectral region. Inset corresponds to the magnified X-ray spectral region at 50 keV and γ-ray spectral region at 511 keV. Band structure, total (black) and projected (color) DOS, of (c) (PEA)_2_PbI_4_, (d) (PEA)_2_MAPb_2_I_7_, (e) (BA)_2_PbI_4_, and (f) (BA)_2_MAPb_2_I_7_ crystals. The Pb p, PEA p, BA p, MA p, and I p are represented by blue, orange, pink, green, and red lines, respectively.

Powder X-ray diffraction (XRD) patterns of ground perovskite crystals are shown in Figure 2a. The prominent low-angle diffraction peaks of A_2_PbX_4_ are indicative of their (002) preferential orientation, while the preferential orientation of A_2_MAPb_2_I_7_ (A = PEA, BA) occurs in the lattice planes of (2–14) and (111), respectively, as displayed in Figure 2a. The XRD patterns of the four crystals were analyzed with the Rietveld refinement method using the FullProf software,^38−41^ and the results are shown in Table 1. Diffractograms of (PEA)_2_PbI_4_, (PEA)_2_MAPb_2_I_7_, (BA)_2_PbI_4_, and (BA)_2_MAPb_2_I_7_ including photographs of the corresponding crystals are shown in Figure S1. The triclinic phase was found with *P*1 space group for (PEA)_2_PbI_4_ and *P*1 for (PEA)_2_MAPb_2_I_7_. The (PEA)_2_MAPb_2_I_7_ single crystals mostly represented a triclinic lattice structure at RT.^40,41^ On the other hand, the orthorhombic phase can be found with primitive centrosymmetric *Pbca* space group for (BA)_2_PbI_4_^42^ and *Cc*2*m* space group for (BA)_2_MAPb_2_I_7_.^42,37,43^ Due to the larger size of iodide than bromide, the (PEA)_2_PbI_4_ crystal shows 209.60 Å^3^ and the (BA)_2_PbI_4_ crystal shows 219.14 Å^3^ larger volume compared to their corresponding bromide crystal. The volume for (PEA)_2_MAPb_2_I_7_ crystal is 378.95 Å^3^ larger than the (BA)_2_MAPb_2_I_7_ crystal, which is due to the larger size of PEA compared to the BA cation.

**Table 1:** Summary of the Crystal Data, Structure Refinements, Bandgaps, and Other Parameters for (PEA)_2_PbI_4_, (PEA)_2_MAPb_2_I_7_, (BA)_2_PbI_4_, and (BA)_2_MAPb_2_I_7_ Crystals at 298 K^a^

	(PEA)_2_PbI_4_	(PEA)_2_MAPb_2_I_7_	(BA)_2_PbI_4_	(BA)_2_MAPb_2_I_7_
empirical formula	C_16_H_24_N_2_PbI_4_	C_17_H_30_N_3_Pb_2_I_7_	C_8_H_24_N_2_PbI_4_	C_9_H_30_N_3_Pb_2_I_7_
formula weight	959.17	1579.12	862.90	1483.04
crystal system	triclinic	triclinic	orthorhombic	orthorhombic
space group	*P*1	*P*1	*Pbca*	*Cc*2*m*
*a* (Å)	8.5835	8.8015	8.2950	8.9785
*b* (Å)	8.6833	8.8130	9.2310	39.4140
*c* (Å)	33.2053	45.7276	27.6290	8.8524
α (deg)	85.1511	97.0364	90.0000	90.0000
β (deg)	85.1281	93.9577	90.0000	90.0000
γ (deg)	90.3921	90.1823	90.0000	90.0000
*V* (Å^3^)	2456.8819	3511.6297	2115.5841	3132.6751
*Z*	4	4	4	4
ρ (g/cm^3^)	2.59	3.00	2.73	3.14
*E*_g_^abs^ (eV)	2.35	2.05	2.41	1.97
*E*_g_^cal^ (eV)	2.13	2.10	2.24	1.80
l at 50 keV (cm)	0.050	0.039	0.042	0.035
l at 511 keV (cm)	3.93	3.22	3.59	3.02
PL peak (eV)	2.35	2.15	2.39	2.13

a The term absorption length is denoted as .

Figure 2b presents the calculated absorption lengths^44^ for photon energies up to 511 keV, and the insets correspond to the magnified X-ray spectral region at 50 keV and γ-ray spectral region at 511 keV. The mass density (ρ) of (PEA)_2_PbI_4_, (PEA)_2_MAPb_2_I_7_, (BA)_2_PbI_4_, and (BA)_2_MAPb_2_I_7_ crystals are calculated as 2.59, 3.00, 2.73, and 3.14 g cm^–3^, respectively.^32,45^ Iodide (I^–^) HOIP crystals have higher ρ than (PEA)_2_PbBr_4_ (2.28 g cm^–3^) or (BA)_2_PbBr_4_ (2.36 g cm^–3^) crystals, respectively.^46^ The larger atomic size of iodide compared to bromide shows high mass density. In our works, ρ values of (PEA)_2_MAPb_2_I_7_ and (BA)_2_MAPb_2_I_7_ crystals are similar in comparison to the reported 3.00 g cm^–3^ ^40^ and 3.16 g cm^–3^,^37^ respectively. As a result, all absorption lengths () at 50 keV of those iodide crystals are at least 50% shorter than those of bromides ( of (BA)_2_PbBr_4_ is 0.099 cm).^46^ However, those  values become at least 5% shorter at 511 keV.^46^ The (BA)_2_MAPb_2_I_7_ has the shortest absorption length of all the studied crystals as can be seen from Figure 2b. At 50 keV, which is widely used in X-ray imaging,  of (PEA)_2_PbI_4_ is 0.050 cm, 16% longer than  of (BA)_2_PbI_4_ of 0.042 cm, and for (PEA)_2_MAPb_2_I_7_ is 0.039 cm, just 11% longer than  of (BA)_2_MAPb_2_I_7_ of 0.035 cm. The effect of the PEA cation on the density leads to a 20% longer  for A_2_PbI_4_ and 6% shorter  for A_2_MAPb_2_I_7_ compared to their BA cation crystals at 511 keV, and the results are summarized in Table 1. For TOF PET, longer  values of (PEA)_2_PbI_4_ among the four crystals are still 44% longer at 50 keV and 50% longer at 511 keV than that of a commercial scintillator, CsI:Tl.^20,24^

The ab initio components for the scintillation efficiencies can be studied through their band structures. Therefore, we employ the density functional theory (DFT) method to calculate the density of states (DOS) and determine the optical bandgap (*E*_g_). The band structures with their total (black) and projected (color) DOS of the studied perovskite crystals are shown in Figures 2c–f. From the calculations, the band structures of all iodide-based QW HOIPS show direct bandgap characteristics.

**Figure 3:**
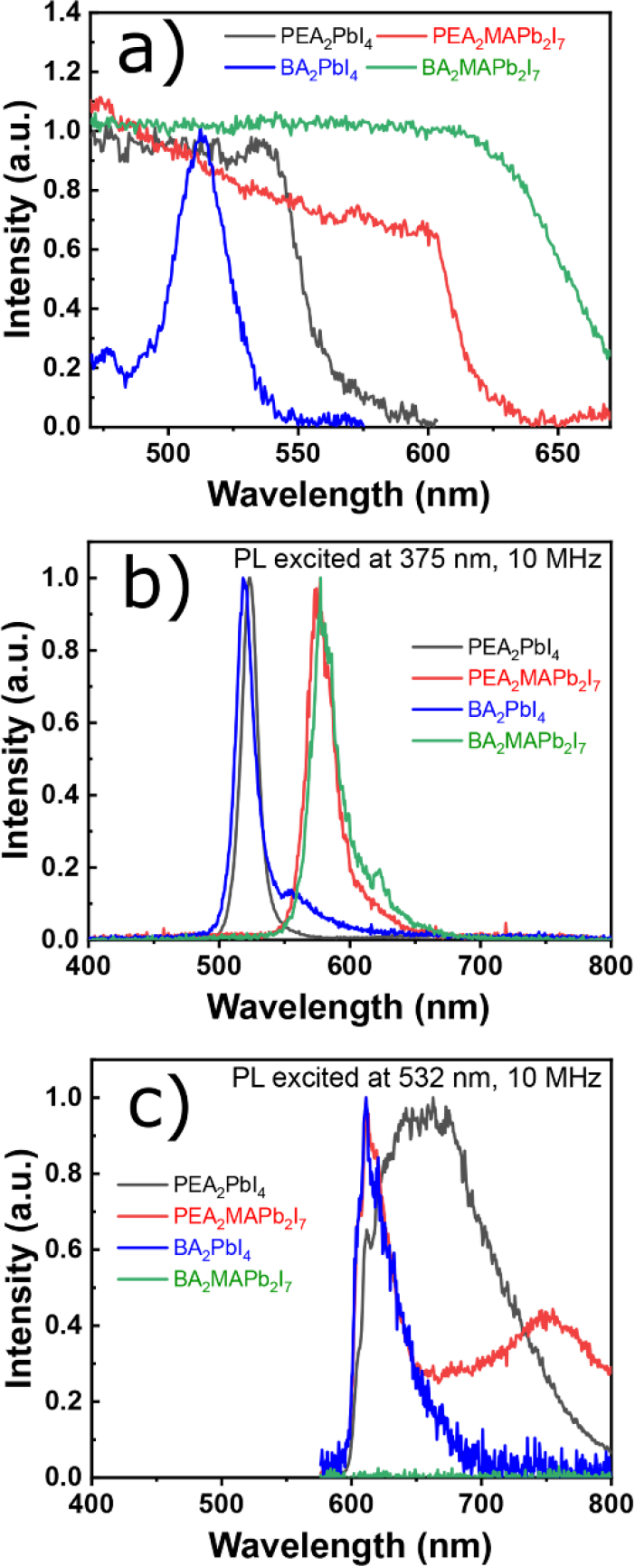
(a) Absorption and PL spectra of (PEA)_2_PbI_4_, (PEA)_2_MAPb_2_I_7_, (BA)_2_PbI_4_, and (BA)_2_MAPb_2_I_7_ perovskites recorded at room temperature (RT) and excited at (b) 375 nm and (c) 532 nm.

We obtained the calculated bandgaps (*E*_g_^cal^) of 2.13, 2.10, 2.24, and 1.80 eV for (PEA)_2_PbI_4_, (PEA)_2_MAPb_2_I_7_, (BA)_2_PbI_4_, and (BA)_2_MAPb_2_I_7_, respectively; the former of (PEA)_2_PbI_4_ shows similar reported values of 2.13 eV,^40,32^ the (BA)_2_PbI_4_ shows similar reported values of 2.28 eV,^47^ and the latter of (PEA)_2_MAPb_2_I_7_ shows smaller than reported values of 2.31 eV.^48^ To compare *E*_g_^cal^, we performed absorption measurements while their spectra and their corresponding bandgap fitting curves with Elliot formalism^49^ are shown in Figure 3a andFigure S2, respectively. On one hand, we obtained small differences of 0.22 and 0.31 eV between *E*_g_^cal^ and calculated Elliot fit bandgaps (*E*_g_^abs^) of 2.35 and 2.41 eV for (PEA)_2_PbI_4_, and (BA)_2_PbI_4_, respectively. On the other hand, we obtained 2.05 and 1.97 eV calculated from Elliot fit for (PEA)_2_MAPb_2_I_7_ and (BA)_2_MAPb_2_I_7_ crystals, respectively. The bandgap of 2.05 eV for the (PEA)_2_MAPb_2_I_7_ crystal is larger compared to reported one of 1.86 eV,^40^ and the bandgap of 1.97 eV for the crystal is coherent with the reported value of 1.99 eV.^27^

PL measurements were performed on bulk crystals of (PEA)_2_PbI_4_, (PEA)_2_MAPb_2_I_7_, (BA)_2_PbI_4_, and (BA)_2_MAPb_2_I_7_ (Figure 3b).^49^ In addition, absorption and PL spectra excited at 375 nm with a logarithmic scale of *y*-axis recorded at RT, decay curves, and photographs of the corresponding (PEA)_2_PbBr_4_ and (BA)_2_PbBr_4_ crystals are shown in Figure S3. PL spectra recorded at RT in Figures 3b,c exhibit two different emission origins depending on the excitation wavelength. On one hand, exciting the samples with 375 nm wavelength produces an emission band at 532 nm (green) for (PEA)_2_PbI_4_ and (BA)_2_PbI_4_ and 577 nm (yellow) for (PEA)_2_MAPb_2_I_7_, and (BA)_2_MAPb_2_I_7_ crystals which is much more intense than the band at 620 nm (red). On the other hand, when using the longer wavelength excitation of 532 nm, only one red emission band is observed at 620 nm (red) for (BA)_2_PbI_4_ and a broad band at 660 nm (red) for (PEA)_2_PbI_4_. For (PEA)_2_MAPb_2_I_7_ crystals, the red emission at 620 nm at its bandgap energy (2.0 eV) with an appreciable PL emission broad band at 748 nm can be originated from the edges of the exfoliated layers of perovskite crystal, as reported by Blancon et al.^50^ The broadband of the PEA_2_PbI_4_ crystal at 660 nm is possibly due to the radiative path of electron capture at a positive iodide vacancy with a subsequent hole capture.^51^ There is no emission band observed for the (BA)_2_MAPb_2_I_7_ crystal at 532 nm excitation wavelength. The green emission band has a full width at half-maximum (FWHM) equal to 19 nm for (PEA)_2_PbI_4_ and (BA)_2_PbI_4_ and 22 nm for (PEA)_2_MAPb_2_I_7_ and (BA)_2_MAPb_2_I_7_ while the red band is broad (110 nm) for (PEA)_2_PbI_4_ and narrow (32 nm) for (BA)_2_PbI_4_ and (PEA)_2_MAPb_2_I_7_. The PL characteristics are observed for (PEA)_2_PbI_4_ and (PEA)_2_MAPb_2_I_7_ crystals since they were synthesized using PbI_2_ precursor and additionally treated with PEAI.^38^ The origin of the green emission band is the characteristic excitonic emission from inorganic PbI_2_ layers while the red emission is associated with the in-plane iodide vacancy causing surface states.^38^

**Figure 4:**
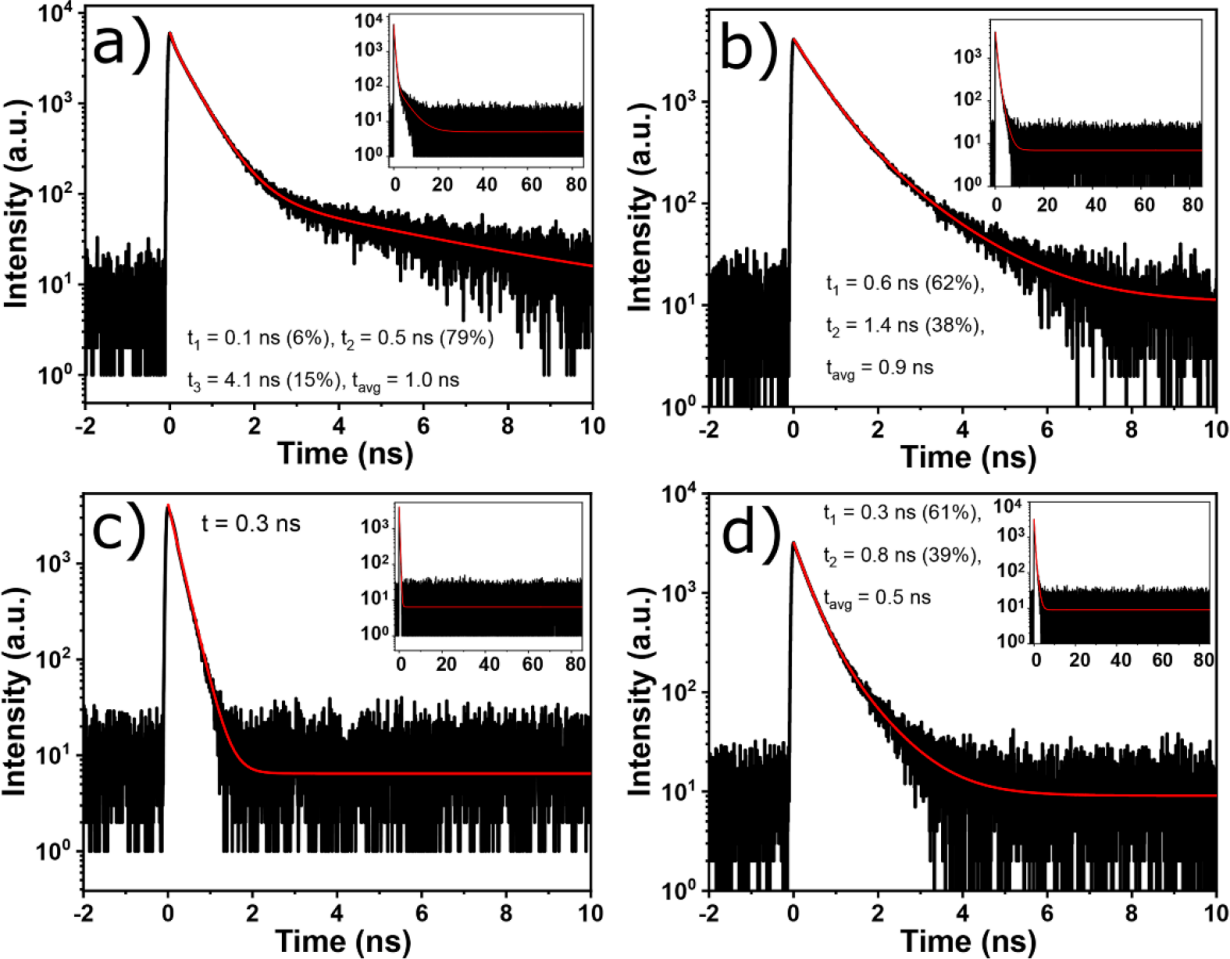
TRPL decay curve excited at 375 nm monitoring 532 nm emission of (a) (PEA)_2_PbI_4_ and (c) (BA)_2_PbI_4_ and 577 nm emission of (b) (PEA)_2_MAPb_2_I_7_ and (d) (BA)_2_MAPb_2_I_7_ crystals. The fits are shown with red lines while the insets correspond to longer time scales.

We probe the spectral origin of the emitting states observed using PL and time-resolved PL (TRPL) spectroscopy. The TRPL decay curves of perovskite crystals were fitted by exponential decay functions which are shown in Figures 4a–d. Most decay times in iodide QW HOIP crystals are faster than those in bromide QW HOIP crystals (see Figure S3), as they can affect the scintillation decay times. Such <1 ns fast decay components of iodide QW HOIP crystals were also observed in previous observations of QW HOIPs.^16,22,32^ The microsecond decay components were also observed under two-photon excitation.^52^ To do the analysis, we also present RT decay curves excited at 532 nm monitoring 620 nm emission in Figure S4. The decay components for the 532 nm emission band of the (PEA)_2_PbI_4_ crystal are 0.1, 0.5, and 4.1 ns, and they are associated with free-exciton emission. The average lifetime value τ_avg_^PL^ of 1.0 ns is similar to those reported in ref (38). In the same crystal, the decay components at 620 nm emission are 3.6 and 37.7 ns, while τ_avg_^PL^ is 36.6 ns. This is 36.6 times slower than τ_avg_^PL^ at 532 nm (see Figure S4). The fastest decay time of 0.3 ns was observed at 532 nm and 0.4 ns at 620 nm for the (BA)_2_PbI_4_ crystal, which are about 4 and 99 times faster than τ_avg_^PL^ at 532 and 620 nm of the (PEA)_2_PbI_4_ crystal, respectively. On the other hand, the decay components for the (PEA)_2_MAPb_2_I_7_ crystal of 0.6 and 1.4 ns correspond to the exciton emission as the decay curve was measured for the 577 nm emission band and τ_avg_^PL^ of 0.9 ns, while the decay components at the 620 nm emission peak position are 0.4 and 7.2 ns, and τ_avg_^PL^ is 4.5 ns, which is 5 times slower than τ_avg_^PL^ at 577 nm emission. The fast decay components for the (BA)_2_MAPb_2_I_7_ crystal of 0.3 and 0.8 ns correspond to the exciton emission as the decay curve was measured for the 577 nm emission band only, and τ_avg_^PL^ of 0.5 ns is observed. Although for (PEA)_2_PbI_4_ and (PEA)_2_MAPb_2_I_7_ crystals the decay times at 577 nm emission are fast, the presence of the emission for respective emission bands at 660 and 748 nm in Figure 3c can make the scintillation decay curves slower as the TRPL monitoring 620 nm emission exhibits slower decay components >8 ns (see Figure S4). As expected from the absence of the long wavelength band in the BA_2_PbI_4_ crystal in Figure 3c, the decay time at 620 nm also yields a similar value as that at 532 nm (see Figures 4c and S4c for comparison).

**Figure 5:**
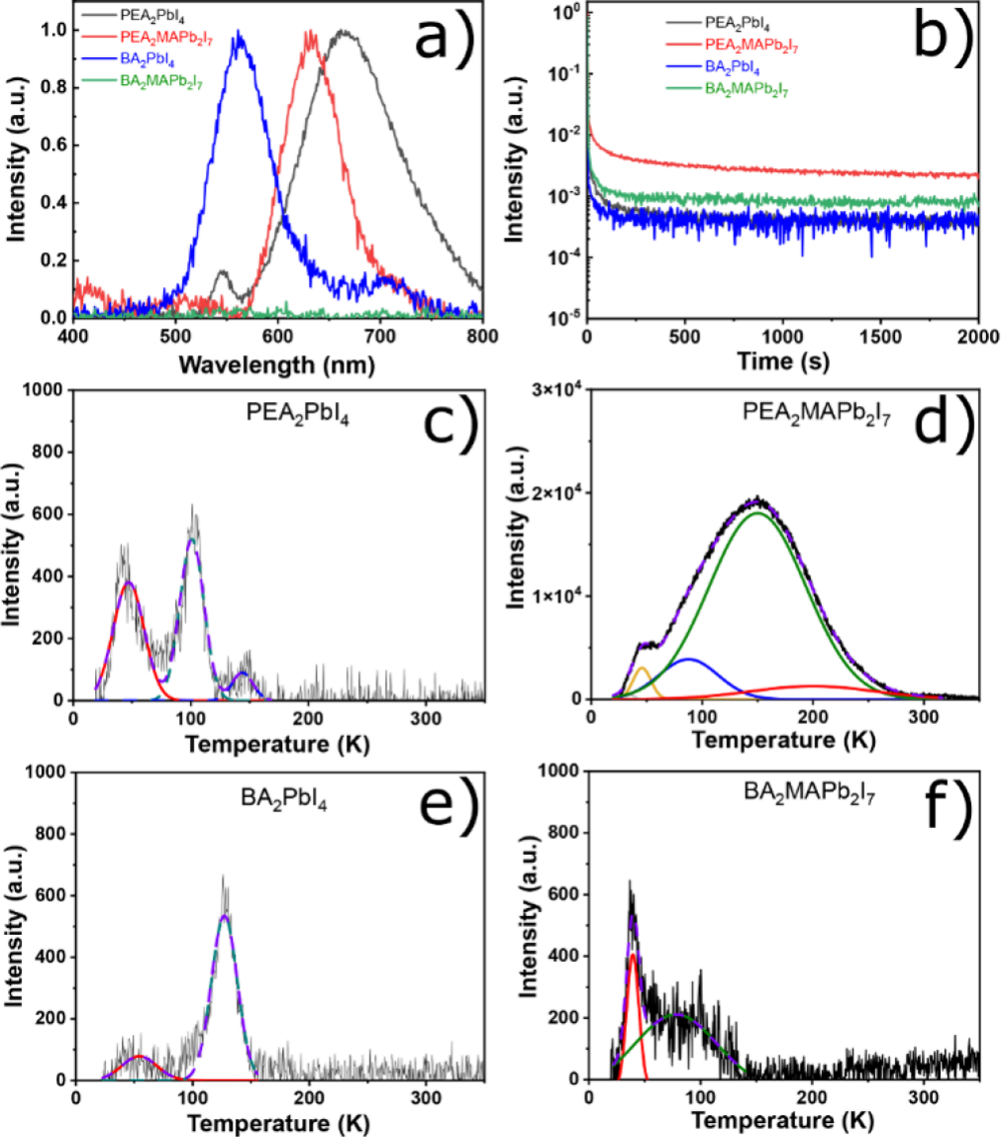
(a) RL spectra at RT. (b) Afterglow decay curves at 10 K of (PEA)_2_PbI_4_, (PEA)_2_MAPb_2_I_7_, (BA)_2_PbI_4_, and (BA)_2_MAPb_2_I_7_ crystals. Afterglow parts were recorded after 10 min of X-ray irradiation. TL spectra and corresponding fits of (c) (PEA)_2_PbI_4_, (d) (PEA)_2_MAPb_2_I_7_, (e) (BA)_2_PbI_4_, and (f) (BA)_2_MAPb_2_I_7_ crystals.

The radioluminescence (RL) spectra at RT in Figure 5a are dominated by the red broadband emission, which resembles the red emission band in PL spectra excited with 532 nm wavelength in Figure 3a for (PEA)_2_PbI_4_ and (PEA)_2_MAPb_2_I_7_ crystals.On one hand, the red surface defect emission (630–665 nm) dominates against the green exciton emission (520–545 nm) for (PEA)_2_PbI_4_ and (PEA)_2_MAPb_2_I_7_ crystals as seen in their RL spectra at RT. This is due to self-absorption as it was observed in other previous QW HOIP crystals.^16,53^ On the other hand, the green surface defect emission (560 nm) strongly dominates the red emission (700 nm) for the (BA)_2_PbI_4_ crystal, and there is no emission excited by X-rays for the (BA)_2_MAPb_2_I_7_ crystal. However, the (PEA)_2_PbI_4_ crystal shows higher self-absorption compared to the (BA)_2_PbI_4_ crystal, and overall the self-absorption of the (PEA)_2_MAPb_2_I_7_ crystal is much more stronger among all the crystals due to presence of MAPbI_3_ impurities.^48^ Afterglow decays recorded after 10 min of X-ray irradiation at the 10 K curve of (PEA)_2_PbI_4_, (PEA)_2_MAPb_2_I_7_, (BA)_2_PbI_4_, and (BA)_2_MAPb_2_I_7_ crystals are shown in Figure 5b. The details of afterglow decay components parameters are reported in Table S1. The afterglow decay components for (PEA)_2_PbI_4_ are 4.9 s (10%), 33.1 s (32%), and 364.3 s (58%), and the average value of afterglow times τ_avg_^afterglow^ is 224.1 s, while for the (BA)_2_PbI_4_ crystal they are 0.2 s (2%), 15.7 s (26%), and 166.5 s (72%) with a τ_avg_^afterglow^ of 87.4 s. On the other hand, the afterglow decay components for (PEA)_2_MAPb_2_I_7_ crystal are 9.6 s (1%), 121.6 s (4%), and 4560.1 s (95%), with τ_avg_^afterglow^ of 4336.5 s, while for the (BA)_2_MAPb_2_I_7_ crystal are 6.5 s (35%), and 66.5 s (65%), with τ_avg_^afterglow^ of 45.4 s. The fastest afterglow is observed for (BA)_2_PbI_4_, which is 5 times faster than (PEA)_2_PbI_4_. The afterglow for A_2_PbI_4_ crystals is faster than that observed in A_2_BPb_2_I_7_ crystals due to low trap density. The presence of traps is directly related to the chain length of the organic cation.^54^ We examine the presence of trap states in the investigated scintillators by performing thermoluminescence (TL) measurements. TL is the phenomenon of afterglow with temperature of a previously exposed materials by high-energy radiation. Originally the thermally activated afterglow is due to the phonon-assisted release of trapped charge carriers with temperature, leading to radiative recombination.^16,33^

TL spectra and the corresponding fits are shown in Figures 5c–f, and TL parameters are given in Table S2 for (PEA)_2_PbI_4_, (PEA)_2_MAPb_2_I_7_, (BA)_2_PbI_4_, and (BA)_2_MAPb_2_I_7_. All samples show several glow peaks as the temperature of the sample is raised, indicating the presence of traps in the crystals. Unfortunately, those traps are mostly deeper, and they have more trapped charge carriers than those in (PEA)_2_PbBr_4_ and (BA)_2_PbBr_4_.^46^ The glow peaks for the (PEA)_2_PbI_4_ crystal are observed at temperatures 47, 101, and 144 K with trap densities of 1.2 × 10^4^, 1.3 × 10^4^, and 2.1 × 10^3^, respectively, as shown in Figure 5c. Significant and several glow peaks with less noisy result for (PEA)_2_MAPb_2_I_7_ crystal are observed due to the high intensity and more traps as summarized in Table S2, including a deep trap over a long temperature range (up to 200 K), as shown in Figure 5d. On the other hand, negligible glow peaks are observed at temperatures 54 and 127 K with trap density of 2.9 × 10^3^ and and 1.5 × 10^4^, respectively, for the (PEA)_2_PbI_4_ crystal as shown in Figure 5e and at 39 and 78 K with trap densities of 1.1 × 10^4^ and 5.5 × 10^3^, respectively, as shown in Figure 5f. A_2_MAPb_2_I_7_ HOIP crystals and especially for the (PEA)_2_MAPb_2_I_7_ crystal show more traps due to the presence of MAPbI_3_ impurities^48^ which has strong traps.^55^ In addition, the (PEA)_2_MAPb_2_I_7_ crystal has traps over a wide range of temperature from 30 to 250 K.

**Figure 6:**
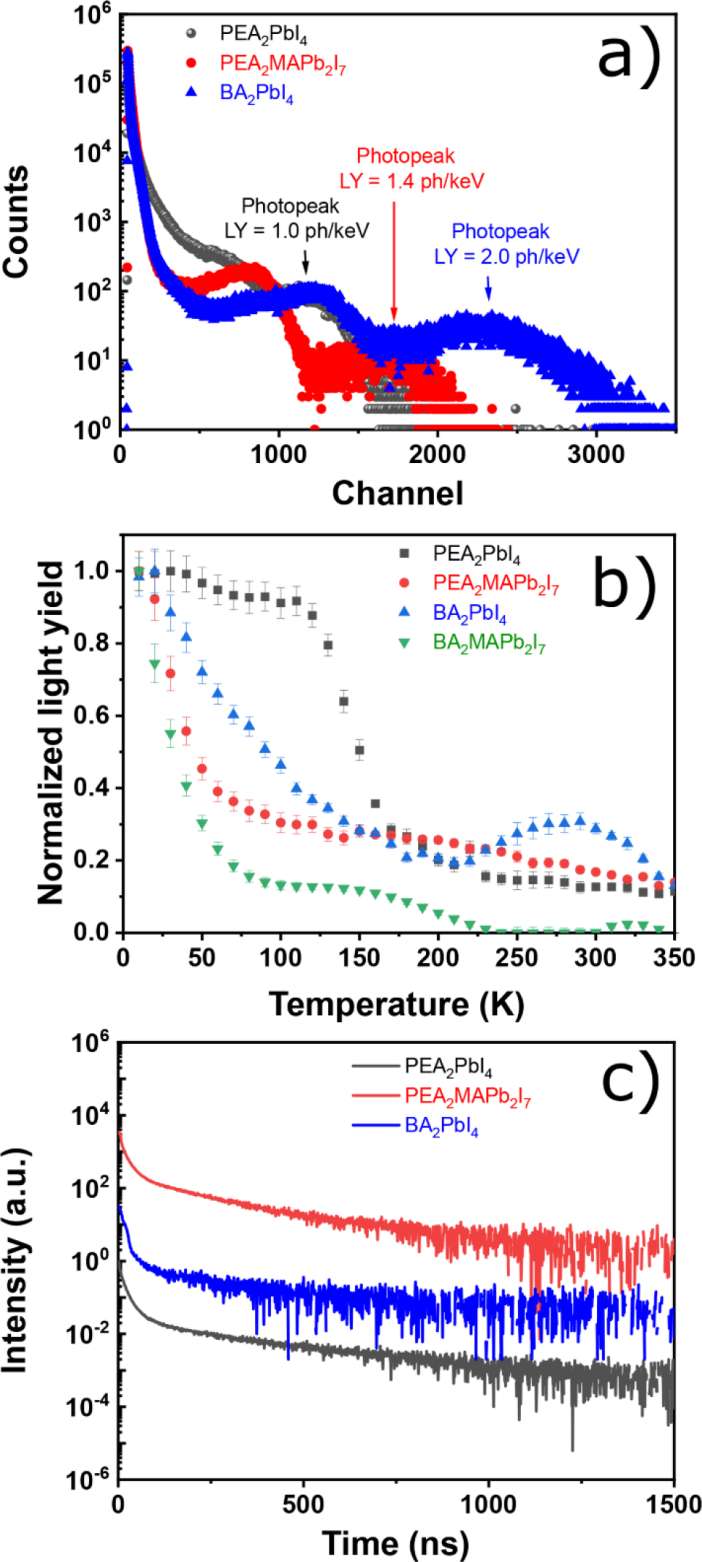
(a) Integrated pulse height in the logarithmic scale. (b) Temperature-dependent light yield from 10 to 350 K under 45 keV X-ray excitation of (PEA)_2_PbI_4_, (PEA)_2_MAPb_2_I_7_, (BA)_2_PbI_4_, and (BA)_2_MAPb_2_I_7_ crystals. (c) Scintillation decay curves of (PEA)_2_PbI_4_, (PEA)_2_MAPb_2_I_7_, and (BA)_2_PbI_4_. For the γ-ray source a ^137^Cs emitting at 662 keV was used.

Light yield is an important property of a scintillator, i.e., the efficiency of the scintillator to convert the energy of absorbed X- and γ-rays into visible photons.^20^ RL measurements were used to determine the comparative values of light yields for perovskite scintillators.^19,56^ The γ-ray pulse height method gives quantitative values for the light yield and also provides information on the energy resolution of the scintillator. We note that the pulse height method is integrated for light yields faster than 2 μs while the RL comparison is integrated over ∼1s longer time. In our current analysis, the light yield is the comparison of the photopeak signals in the pulse height spectra at certain energy of γ-ray radiation with the scintillator single electron response.^33^Figure 6a present the results for pulse height spectra recorded under γ-ray excitation. The pulse height spectrum in Figure 6a for (PEA)_2_PbI_4_, (BA)_2_PbI_4_, and (PEA)_2_MAPb_2_I_7_ crystals exhibit structures of Compton scattering and photoelectric peak; however, the obtained energy resolution value at 662 keV from the ^137^Cs source of γ-ray excitation is above 32%, which is still far from beating the best energy resolution for a lithium-doped (PEA)_2_PbBr_4_ of 7.7% at the same energy.^16^Figure 6b shows the temperature-dependent normalized light yield under 45 keV X-ray excitation.

Based on the integration of the RL intensities at each temperature, the temperature-dependent light yield was calculated. Under X-ray excitation, large amounts of charge carries are involved, leading to the large possibility of trapping.^16^ As shown in Figure S5, the temperature-dependent RL 2D maps of some HOIP crystals illustrate their different patterns. Because of the negative thermal quenching behaviors, bromide HOIP crystals have maximum light yields at temperatures close to RT,^16^ while iodide HOIP crystals suffer from the regular thermal quenching, so their light yields at RT is not as high as those at low temperatures. At 10 K, all samples exhibit the maximum light yields which then gradually decrease by temperature raising. By comparing the organic cation of A_2_PbI_4_ or A_2_MAPb_2_I_7_ HOIP crystals with the same iodide, for instance, (PEA)_2_PbI_4_ and (BA)_2_PbI_4_ or (PEA)_2_MAPb_2_I_7_ and (BA)_2_MAPb_2_I_7_, it is evident that PEA cation ones have higher light yields. To discuss the difference, the light yield (LY), expressed in photons/keV, is given by the relation
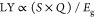
1where *S* denotes the electron–hole transport efficiency to the exciton recombination and *Q* signifies the luminescence quantum efficiency of the exciton. There are also losses during the transport of the light in the detector due to internal scattering and reabsorption, so the actual light yield of the scintillator might be less than expected, depending on the geometry of the scintillator.^20^ The light yields of all QW HOIPS are summarized in Table S3. (BA)_2_PbI_4_ has the highest light yield of 2 photons/keV among all crystals while (PEA)_2_PbI_4_ has 1 photon/keV at RT. The light yield of (BA)_2_PbI_4_ has improved at both RT and 10 K compared to the reported^16^ one as shown in Table S3 due to the different fabrication method. Because (BA)_2_PbI_4_ has a slightly larger *E*_g_ of 0.1 eV, we expect that the light yield is to be smaller, but it appears that (BA)_2_PbI_4_ has a larger *Q* and/or *S* than (PEA)_2_PbI_4_. This can be seen in the trend between (BA)_2_PbBr_4_ and (PEA)_2_PbBr_4_.^53,33^ For (PEA)_2_MAPb_2_I_7_, the light yield is slightly improved than that of (PEA)_2_PbI_4_ because the bandgap is smaller (2.10 eV). The smallest bandgap for (BA)_2_MAPb_2_I_7_ is 1.80 eV and it shows a very small light yield at RT (see also Figure S5) as there is a strong quenching due to the bandgap being too small.^57^ The maximum light yield of (PEA)_2_PbBr_4_ crystal at RT is 10 photons/keV,^58,59^ larger than the iodide-based QW HOIP crystal scintillators. On one hand, the light yield for the (PEA)_2_MAPb_2_I_7_ crystal is 1.4 photons/keV at RT, which is still low compared to the (BA)_2_PbI_4_ HOIP scintillator of 2 photons/keV. On the other hand, the (PEA)_2_MAPb_2_I_7_ scintillator has a peak position in the pulse height spectrum which is slightly higher than the position in that of the (PEA)_2_PbI_4_ scintillator meaning higher light yield. Because the light yields at RT were observed in three crystals, the delay distribution and the coincidence timing resolution (CTR) measurement results for (PEA)_2_PbI_4_, (BA)_2_PbI_4_, and (PEA)_2_MAPb_2_I_7_ crystals are shown in ref (32) and Figure S6, respectively. We obtain CTR values of 138 ± 5, 149 ± 10, and 207 ± 14 ps for (PEA)_2_PbI_4_, (BA)_2_PbI_4_, and (PEA)_2_MAPb_2_I_7_ crystals, respectively, and all still have similar values. They still can be 2 times improved by increasing the light yield at low temperature, making them similar to or slightly better than those of bromide crystals.^32^ The light yield stability measured from the pulse height spectra of the (PEA)_2_MAPb_2_I_7_ crystal for 6 h and the derived values of the light yield were plotted with the normalized values at the initial time as shown in Figure S7, showing that the hygroscopicity is not as notorious as other iodides, e.g., LaI_3_:Ce^3+^.^57^ We determine the decay at high energies by investigating the γ-ray excited scintillation decay curves recorded under γ-ray excitation at 662 keV presented in Figure 6c while we report the exponential fitting parameters for the decay curves in Table S4. From the decay curves in Figure 6c, we can immediately see that (BA)_2_PbI_4_ scintillator leads to a faster decay compared to the (PEA)_2_PbI_4_ scintillator, while the decay of (PEA)_2_MAPb_2_I_7_ is the slowest. The QW HOIP samples show average decay times of 190 and 111 ns for (PEA)_2_PbI_4_ and (BA)_2_PbI_4_, respectively. The fastest decay components of the QW HOIP crystals are 0.5 and 0.4 ns for (PEA)_2_PbI_4_ and (BA)_2_PbI_4_, respectively. Such ultrafast decay components can be linked to TRPL decay curves in Figures 4 and S4, and they are not observed in (PEA)_2_PbBr_4_ and (BA)_2_PbBr_4_.^46^ The (PEA)_2_MAPb_2_I_7_ perovskite shows an average decay time of 112 ns and decay components of 9.3 ns (37%), 43 ns (23%), and 249 ns (39%). The fast component (<1 ns) is not observed for the (PEA)_2_MAPb_2_I_7_ perovskite due to the different scintillation mechanism, dominant slow exciton, and absence of free exciton emission in RL at RT. It is possible that the average lifetimes <200 ns observed are due to the higher presence of traps that lead to nonradiative processes and thus lead to a faster decay of the luminescence.^33^

## Conclusions

In summary, we investigated the effects of PEA and BA cation on the crystal structure as well as optical and scintillation properties of both QW HOIP A_2_PbI_4_ and A_2_MAPb_2_I_7_ crystals based on the XRD analysis, absorption, PL, TRPL, temperature-dependent RL, and pulse height measurements. Based on the XRD results, we calculated the band structure and density of states using DFT analysis. From the PL measurement, we observed that A_2_PbI_4_ crystals exhibit green and red emission with fastest PL decay time. Overall, we observed that (BA)_2_MAPb_2_I_7_ scintillator exhibits the highest mass density, and smaller bandgap due to the increased thickness of the inorganic slabs. We conducted temperature-dependent RL measurements to explore the effects of temperature on the scintillation properties of the perovskites, including the effect on the afterglow. We observed comparable light yields for all iodide crystals at 10 K (∼10 photons/keV), while light yields at RT (between 1 and 2 photons/keV) are still much lower compared to QW HOIP bromide crystals. The biggest advantages of QW HOIP iodide scintillators compared to their bromide counterparts are shorter radiation (X- and γ-ray) absorption lengths (between 0.035 and 0.050 cm at 50 keV), faster decay time components (between 0.3 and 1.0 ns), and comparable light yields (between 7.5 and 10 photons/keV) at low temperature. Furthermore, our study of optical and scintillation properties of both A_2_PbI_4_ and A_2_MAPb_2_I_7_ crystals provides insight for further improvements on the radiation absorptions and emission rates toward high sensitivity and fast radiation detection applications.

## Supporting Information Available

Rietveld refinement of crystal XRD diffractograms, absorption spectra with Elliot fits, TRPL recorded at 532 nm excitation monitoring 620 nm emission, RL spectra mapping at different temperatures, CTR results, and light yield stability of iodide-based QW HOIPS; PL and absorption spectra of (PEA)_2_PbBr_4_ and (BA)_2_PbBr_4_ (PDF)
Crystallographic data for (PEA)_2_PbI_4_ crystal (CIF)
Crystallographic data for (PEA)_2_MAPb_2_I_7_ crystal (CIF)
Crystallographic data for (BA)_2_PbI_4_ crystal (CIF)
Crystallographic data for (BA)_2_MAPb_2_I_7_ crystal (CIF)

## Supplementary Material

jp3c00824_si_001.pdf

jp3c00824_si_002.cif

jp3c00824_si_003.cif

jp3c00824_si_004.cif

jp3c00824_si_005.cif

## Abbreviations

HOIP: hybrid organic–inorganic perovskites
QW: quantum well
2D: two-dimensional
3D: three-dimensional
PEA: phenethylammonium
BA: butylammonium
RT: room temperature
XRD: X-ray diffraction
DOS: density of states
DFT: density functional theory
PL: photoluminescence
TRPL: time-resolved PL
RL: radioluminescence
TL: thermoluminescence
CTR: coincidence time resolution.

## References

[ref1] Kojima A.; Teshima K.; Shirai Y.; Miyasaka T. Organometal halide perovskites as visible-light sensitizers for photovoltaic cells. J. Am. Chem. Soc. 2009, 131 (17), 6050–6051. 10.1021/ja809598r. 19366264

[ref2] Li P.; Zhang Y.; Liang C.; Xing G.; Liu X.; Li F.; Liu X.; Hu X.; Shao G.; Song Y. Phase pure 2D perovskite for high-performance 2D-3D heterostructured perovskite solar cells. Adv. Mater. 2018, 30, 1805323 10.1002/adma.201805323. 30387210

[ref3] Zuo C. T.; Bolink H. J.; Han H. W.; Huang J. S.; Cahen D.; Ding L. M. Advances in perovskite solar cells. Adv. Sci. 2016, 3, 1500324 10.1002/advs.201500324. PMC5066666 27812475

[ref4] Kim H.-S.; Hagfeldt A.; Park N.-G. Morphological and compositional progress in halide perovskite solar cells. Chem. Commun. 2019, 55, 1192–1200. 10.1039/C8CC08653B. 30604789

[ref5] Wang P.; Wu Y.; Cai B.; Ma Q.; Zheng X.; Zhang W. H. Solution-processable perovskite solar cells toward commercialization: progress and challenges. Adv. Funct. Mater. 2019, 29, 1807661 10.1002/adfm.201807661.

[ref6] Chin X. Y.; Cortecchia D.; Yin J.; Bruno A.; Soci C. Lead iodide perovskite light-emitting field-effect transistor. Nat. Commun. 2015, 6, 7383 10.1038/ncomms8383. 26108967 PMC4491174

[ref7] Smith I. C.; Hoke E. T.; Solis-Ibarra D.; McGehee M. D.; Karunadasa H. I. A layered hybrid perovskite solar-cell absorber with enhanced moisture stability. Angew. Chem., Int. Ed. 2014, 53 (42), 11232–11235. 10.1002/anie.201406466. 25196933

[ref8] Mao L.; Wu Y.; Stoumpos C. C.; Wasielewski M. R.; Kanatzidis M. G. White-light emission and structural distortion in new corrugated two-dimensional lead bromide perovskites. J. Am. Chem. Soc. 2017, 139 (14), 5210–5215. 10.1021/jacs.7b01312. 28306254

[ref9] Correa-Baena J.-P.; Abate A.; Saliba M.; Tress W.; Jesper Jacobsson T.; Gratzel M.; Hagfeldt A. The rapid evolution of highly efficient perovskite solar cells. Energy Environ. Sci. 2017, 10, 710–727. 10.1039/C6EE03397K.

[ref10] Tsai H.; Nie W.; Blancon J.-C.; Stoumpos C. C.; Soe C. M. M.; Yoo J.; Crochet J.; Tretiak S.; Even J.; Sadhanala A.; et al. Stable light-emitting diodes using phase-pure ruddlesden–popper layered perovskites. Adv. Mater. 2018, 30, 1704217 10.1002/adma.201704217. 29314326

[ref11] Sutherland B. R.; Sargent E. H. Perovskite photonic sources. Nat. Photonics 2016, 10, 295–302. 10.1038/nphoton.2016.62.

[ref12] Wang H.; Kim D. H. Perovskite-based photodetectors: materials and devices. Chem. Soc. Rev. 2017, 46, 5204–5236. 10.1039/C6CS00896H. 28795697

[ref13] Fu Y.; Zhu H.; Chen J.; Hautzinger M. P.; Zhu X.-Y.; Jin S. Metal halide perovskite nanostructures for optoelectronic applications and the study of physical properties. Nat. Rev. Mater. 2019, 4, 169–188. 10.1038/s41578-019-0080-9.

[ref14] Xie A.; Hettiarachchi C.; Maddalena F.; Witkowski M. E.; Makowski M.; Drozdowski W.; Arramel; Wee A. T. S.; Springham S. V.; Vuong P. Q.; et al. Lithium-doped two-dimensional perovskite scintillator for wide-range radiation detection. Commun. Mater. 2020, 1 (1), 37 10.1038/s43246-020-0038-x.

[ref15] Kumar S.; Jagielski J.; Yakunin S.; Rice P.; Chiu Y.-C.; Wang M.; Nedelcu G.; Kim Y.; Lin S.; Santos E. J. G.; et al. Efficient blue electroluminescence using quantum-confined two-dimensional perovskites. ACS Nano 2016, 10 (10), 9720–9729. 10.1021/acsnano.6b05775. 27684448

[ref16] Xie A.; Maddalena F.; Witkowski M. E.; Makowski M.; Mahler B.; Drozdowski W.; Springham S. V.; Coquet P.; Dujardin C.; Birowosuto M. D.; et al. Library of two-dimensional hybrid lead halide perovskite scintillator crystals. Chem. Mater. 2020, 32 (19), 8530–8539. 10.1021/acs.chemmater.0c02789.

[ref17] Wibowo A.; Sheikh M. A. K.; Diguna L. J.; Ananda M. B.; Marsudi M. A.; Arramel A.; Zeng S.; Wong L. J.; Birowosuto M. D. Development and challenges in perovskite scintillators for high resolution imaging, and timing applications. Commun. Mater. 2023, 10.1038/s43246-023-00348-5.

[ref18] Yang Y.; Gao F.; Gao S.; Wei S.-H. Origin of the stability of two-dimensional perovskites: a first-principles study. J. Mater. Chem. A 2018, 6, 14949–14955. 10.1039/C8TA01496E.

[ref19] Birowosuto M. D.; Cortecchia D.; Drozdowski W.; Brylew K.; Lachmanski W.; Bruno A.; Soci C. X-ray scintillation in lead halide perovskite crystals. Sci. Rep. 2016, 6 (1), 37254 10.1038/srep37254. 27849019 PMC5111063

[ref20] Maddalena F.; Tjahjana L.; Xie A.; Arramel; Zeng S.; Wang H.; Coquet P.; Drozdowski W.; Dujardin C.; Dang C. Inorganic, organic, and perovskite halides with nanotechnology for high–light yield X- and γ-ray scintillators. Crystals 2019, 9 (88), 88 10.3390/cryst9020088.

[ref21] Kawano N.; Koshimizu M.; Horiai A.; Nishikido F.; Haruki R.; Kishimoto S.; Shibuya K.; Fujimoto Y.; Yanagida T.; Asai K. Effect of organic moieties on the scintillation properties of organic–inorganic layered perovskite-type compounds. Jpn. J. Appl. Phys. 2016, 55, 110309 10.7567/JJAP.55.110309.

[ref22] Shibuya K.; Koshimizu M.; Takeoka Y.; Asai K. Scintillation properties of (C_6_H_13_NH_3_)_2_PbI_4_: exciton luminescence of an organic/inorganic multiple quantum well structure compound induced by 2.0 MeV protons. Nucl. Instrum. Methods Phys. Res., Sect. B 2002, 194 (2), 207–212. 10.1016/S0168-583X(02)00671-7.

[ref23] Kishimoto S.; Shibuya K.; Nishikido F.; Koshimizu M.; Haruki R.; Yoda Y. Subnanosecond time resolved X-ray measurements using an organic-inorganic perovskite scintillator. Appl. Phys. Lett. 2008, 93 (26), 261901 10.1063/1.3059562.

[ref24] Dujardin C.; Auffray E.; Bourret-Courchesne E.; Dorenbos P.; Lecoq P.; Nikl M.; Vasil’ev A. N.; Yoshikawa A.; Zhu R. Y. Needs, trends, and advances in inorganic scintillators. IEEE Trans. Nucl. Sci. 2018, 65 (8), 1977–1997. 10.1109/TNS.2018.2840160.

[ref25] Tsai H.; Nie W.; Blancon J.-C.; Stoumpos C. C.; Asadpour R.; Harutyunyan B.; Neukirch A. J.; Verduzco R.; Crochet J. J.; Tretiak S.; Pedesseau L.; Even J.; Alam M. A.; Gupta G.; Lou J.; Ajayan P. M.; Bedzyk M. J.; Kanatzidis M. G.; Mohite A. D. High-efficiency two-dimensional Ruddlesden–Popper perovskite solar cells. Nature 2016, 536, 312–316. 10.1038/nature18306. 27383783

[ref26] Xiao Z.; Meng W.; Wang J.; Mitzi D. B.; Yan Y. Searching for promising new perovskite-based photovoltaic absorbers: the importance of electronic dimensionality. Mater. Horiz. 2017, 4, 206–216. 10.1039/C6MH00519E.

[ref27] Soe C. M. M.; Stoumpos C. C.; Kepenekian M.; Traore B.; Tsai H.; Nie W.; Wang B.; Katan C.; Seshadri R.; Mohite A. D.; Even J.; Marks T. J.; Kanatzidis M. G. New type of 2D perovskites with alternating cations in the interlayer space, (C(NH_2_)_3_)(CH_3_NH_3_)_n_Pb_n_I_3n+1_: structure, properties, and photovoltaic performance. J. Am. Chem. Soc. 2017, 139 (45), 16297–16309. 10.1021/jacs.7b09096. 29095597

[ref28] Kagan C. R.; Mitzi D. B.; Dimitrakopoulos C. D. D. B. M. a. C. D. D. Organic-inorganic hybrid materials as semiconducting channels in thin-film field-effect transistors. Science 1999, 286, 945 10.1126/science.286.5441.945. 10542146

[ref29] Wu X.; Trinh M. T.; Niesner D.; Zhu H.; Norman Z.; Owen J. S.; Yaffe O.; Kudisch B. J.; Zhu X.-Y. Trap states in lead iodide perovskites. J. Am. Chem. Soc. 2015, 137 (5), 2089–2096. 10.1021/ja512833n. 25602495

[ref30] Yuan M.; Quan L. N.; Comin R.; Walters G.; Sabatini R.; Voznyy O.; Hoogland S.; Zhao Y.; Beauregard E. M.; Kanjanaboos P.; Lu Z.; Kim D. H.; Sargent E. H. Perovskite energy funnels for efficient light-emitting diodes. Nat. Nanotechnol. 2016, 11, 872 10.1038/nnano.2016.110. 27347835

[ref31] Wong J.; Yang K. 2D hybrid halide perovskites: synthesis, properties, and applications. Sol. RRL 2021, 5 (1), 2000395 10.1002/solr.202000395.

[ref32] Kowal D.; Makowski M.; Witkowski M. E.; Cala’ R.; Sheikh Md A. K.; Mahyuddin M. H.; Auffray E.; Drozdowski W.; Cortecchia D.; Birowosuto M. PEA_2_PbI_4_: Fast two-dimensional lead iodide perovskite scintillator with green and red emission. Mater. Today Chem. 2023, 10.1016/j.mtchem.2023.101455.

[ref33] Maddalena F.; Xie A.; Arramel; Witkowski M. E.; Makowski M.; Mahler B.; Drozdowski W.; Mariyappan T.; Springham S. V.; Coquet P.; et al. Effect of commensurate lithium doping on the scintillation of two-dimensional perovskite crystals. J. Mater. Chem. C 2021, 9, 2504–2512. 10.1039/D0TC05647B.

[ref34] Kohn W.; Sham L. J. Self-consistent equations including exchange and correlation effects. Phys. Rev. 1965, 140, A1133–A1138. 10.1103/PhysRev.140.A1133.

[ref35] Kresse G.; Furthmüller J. Efficiency of ab-initio total energy calculations for metals and semiconductors using a plane-wave basis set. Comput. Mater. Sci. 1996, 6 (1), 15–50. 10.1016/0927-0256(96)00008-0.

[ref36] Blöchl P. E. Projector augmented-wave method. Phys. Rev. B 1994, 50, 17953–17979. 10.1103/PhysRevB.50.17953. 9976227

[ref37] Stoumpos C. C.; Cao D. H.; Clark D. J.; Young J.; Rondinelli J. M.; Jang J. I.; Hupp J. T.; Kanatzidis M. G. Ruddlesden-popper hybrid lead iodide perovskite 2D homologous semiconductors. Chem. Mater. 2016, 28, 2852–2867. 10.1021/acs.chemmater.6b00847.

[ref38] Yin J.; Naphade R.; Arzaluz L. G.; Brédas J.-L.; Bakr O. M.; Mohammed O. M. Modulation of broadband emissions in two dimensional ⟨100⟩-oriented ruddlesden-popper hybrid perovskites. ACS Energy Lett. 2020, 5, 2149–2155. 10.1021/acsenergylett.0c01047.

[ref39] Du K.-Z.; Tu Q.; Zhang X.; Han Q.; Liu J.; Zauscher S.; Mitzi D. B. Two-dimensional lead(ii) halide-based hybrid perovskites templated by acene alkylamines: crystal structures, optical properties, and piezoelectricity. Inorg. Chem. 2017, 56, 9291–9302. 10.1021/acs.inorgchem.7b01094. 28749133

[ref40] Song J.; Dang Y.; Liu X. L.; Tao X. Layered hybrid lead perovskite single crystals: phase transformations and tunable optical properties. CrystEngComm 2020, 22, 6310–6315. 10.1039/D0CE00753F.

[ref41] Calabrese J.; Jones N. L.; Harlow R. L.; Herron N.; Thorn D. L.; Wang Y. Preparation and characterization of layered lead halide compounds. J. Am. Chem. Soc. 1991, 113 (6), 2328–2330. 10.1021/ja00006a076.

[ref42] Mitzi D. B. Synthesis, crystal structure, and optical and thermal properties of (C_4_H_9_NH_3_)_2_MI_4_ (M = Ge, Sn, Pb). Chem. Mater. 1996, 8 (3), 791–800. 10.1021/cm9505097.

[ref43] Billing D. G.; Lemmerer A. Synthesis, characterization and phase transitions in the inorganic-organic layered perovskite-type hybrids [(C_n_H_2n+1_NH_3_)_2_PbI_4_], n = 4, 5 and 6. Acta Crystallogr., Sect. B: Struct. Sci. 2007, 63, 735–747. 10.1107/S0108768107031758. 17873443

[ref44] NIST X-ray attenuation calculator. https://physics.nist.gov/PhysRefData/FFast/html/form.html (accessed 2023-01-20).

[ref45] Sheikh Md A. K.; Kowal D.; Mahyuddin M. H.; Onggo D.; Maddalena F.; Dang C.; Cala’ R.; Auffray E.; Witkowski M. E.; Makowski M.; et al. Solution-processable A_2_XY_4_ (A = PEA, BA, X= Pb, Sn, Cu, Mn, Y = Cl, Br, I) crystals for high light yield and ultrafast scintillators. IEEE Trans. Nucl. Sci. 2023, 1 10.1109/TNS.2023.3267636.

[ref46] Maddalena F.; Mahyuddin M. H.; Kowal D.; Witkowski M. E.; Makowski M.; Sheikh M. A. K.; Mahato S.; Jédrzejewski R.; Drozdowski W.; Dujardin C.; Lattice expansion in rubidium doped hybrid organic-inorganic perovskite crystals resulting smaller-bandgap and higher-light-yield scintillators. Inorg. Chem. 2023 (under review). 10.1021/acs.inorgchem.3c00270 PMC10265701 37236171

[ref47] Yuan Y.; Liu X.-F.; Ma X.; Wang X.; Li X.; Xiao J.; Li X.; Zhang H.-L.; Wang L. Large band gap narrowing and prolonged carrier lifetime of (C_4_H_9_NH_3_)_2_PbI_4_ under high pressure. Adv. Sci. 2019, 6, 1900240 10.1002/advs.201900240. PMC6685472 31406664

[ref48] Peng W.; Yin J.; Ho K.-T.; Ouellette O.; De Bastiani M.; Murali B.; El Tall O.; Shen C.; Miao X.; Pan J.; Alarousu E.; He J.-H.; Ooi B. S.; Mohammed O. F.; Sargent E.; Bakr O. M. Ultralow delf-doping in two-dimensional hybrid perovskite single crystals. Nano Lett. 2017, 17, 4759 10.1021/acs.nanolett.7b01475. 28657752

[ref49] Elliott R. J. Theory of the effect of spin-orbit coupling on magnetic resonance in some semiconductors. Phys. Rev. 1954, 96 (2), 266–279. 10.1103/PhysRev.96.266.

[ref50] Blancon J.-C.; Tsai H.; Nie W.; Stoumpos C. C.; Pedesseau L.; Katan C.; Kepenekian M.; Soe C. M. M.; Appavoo K.; Sfeir M. Y.; et al. Extremely efficient internal exciton dissociation through edge states in layered 2D perovskite. Science 2017, 355 (6331), 1288–1292. 10.1126/science.aal4211. 28280250

[ref51] Kahmann S.; Meggiolaro D.; Gregori L.; Tekelenburg E. K.; Pitaro M.; Stranks S. D.; De Angelis F.; Loi M. A. The origin of broad emission in ⟨100⟩ two dimensional perovskites: extrinsic vs intrinsic processes. ACS Energy Lett. 2022, 7, 4232–4241. 10.1021/acsenergylett.2c02123. 36531144 PMC9745793

[ref52] Fang H.-H.; Yang J.; Adjokatse S.; Tekelenburg E.; Kamminga M. E.; Duim H.; Ye J.; Blake G. R.; Even J.; Loi M. A. Band-edge exciton fine structure and exciton recombination dynamics in single crystals of layered hybrid perovskites. Adv. Funct. Mater. 2020, 30, 1907979 10.1002/adfm.201907979.

[ref53] Diguna L. J.; Jonathan L.; Mahyuddin M. H.; Arramel; Maddalena F.; Mulyani I.; Onggo D.; Bachiri A.; Witkowski M. E.; Makowski M.; et al. BA_2_XBr_4_ (X = Pb, Cu, Sn): from lead to lead-free halide perovskite scintillators. Mater. Adv. 2022, 3 (12), 5087–5095. 10.1039/D2MA00258B.

[ref54] Hardhienata H.; Ahmad F.; Aminah M.; Onggo D.; Diguna L. J.; Birowosuto M. D.; Witkowski M. E.; Makowski M.; Drozdowski W. Optical and X-ray scintillation properties of X_2_MnCl_4_ (X= PEA, PPA) perovskite crystals. J. Phys. D: Appl. Phys. 2020, 53 (45), 455303 10.1088/1361-6463/aba461.

[ref55] Gordillo G.; Otálora C. A.; Ramirez A. A. A study of trap and recombination centers in MAPbI_3_ perovskites. Phys. Chem. Chem. Phys. 2016, 18, 32862–32867. 10.1039/C6CP06261J. 27883123

[ref56] Zhai W.; Ge C.; Fang X.; Zhang K.; Tian C.; Yuan K.; Sun S.; Li Y.; Chen W.; Ran G. Acetone vapour-assisted growth of 2D single-crystalline organic lead halide perovskite microplates and their temperature-enhanced photoluminescence. RSC Adv. 2018, 8, 14527–14531. 10.1039/C8RA00583D. 35540773 PMC9079928

[ref57] Bessiere A.; Dorenbos P.; van Eijk C. W. E.; Kramer K. W.; Gudel H. U.; de Mello Donega C.; Meijerink A. Luminescence and scintillation properties of the small band gap compound LaI_3_:Ce^3+^. Nucl. Instrum. Methods Phys. Res., Sect. A 2005, 537 (1–2), 22–26. 10.1016/j.nima.2004.07.224.

[ref58] van Blaaderen J. J.; Maddalena F.; Dang C.; Birowosuto M. D.; Dorenbos P. Temperature dependent scintillation properties and mechanisms of (PEA)_2_PbBr_4_ single crystals. J. Mater. Chem. C 2022, 10, 11598–11606. 10.1039/D2TC01483A. PMC9386685 36090966

[ref59] Cala’ R.; Frank I.; Pagano F.; Maddalena F.; Dang C.; Birowosuto M. D.; Auffray E. Sub-100-ps time resolution from undoped and Li-doped two-dimensional perovskite scintillators. Appl. Phys. Lett. 2022, 120 (24), 241901 10.1063/5.0093606.

