# Supplementary material for: A2Bn–1PbnI3n+1 (A = BA, PEA; B = MA; n = 1, 2): Engineering Quantum-Well Crystals for High Mass Density and Fast Scintillators
Source: J Phys Chem C Nanomater Interfaces. 2023 Apr 26;127(22):10737–47. doi: 10.1021/acs.jpcc.3c00824 (PMC10258843; doi:10.1021/acs.jpcc.3c00824)
Supplement: Supplementary file 1 — jp3c00824_si_001.pdf [file jp3c00824_si_001.pdf]

$A_2B_{n-1}Pb_nI_{3n+1}$ (A = BA, PEA; B = MA, n = 1, 2): Engineering Quantum-well Crystals for High Mass Density and Fast Scintillators

Md Abdul Kuddus Sheikh[1], Dominik Kowal[1], Muhammad Haris Mahyuddin[2], Roberto Cala'[3,4], Etiennette Auffray[4], Marcin Eugeniusz Witkowski[5], Michal Makowski[5], Winicjusz Drozdowski[5], Hong Wang[6], Christophe Dujardin[7], Daniele Cortecchia[8,*] Muhammad Danang Birowosuto[1,*]

[1] Łukasiewicz Research Network-PORT Polish Center for Technology Development, Stabłowicka 147, Wrocław, 54-066, Poland;

[2] Research Group of Advanced Functional Materials and Research Center for Nanoscience and Nanotechnology, Institut Teknologi Bandung, Jl. Ganesha 10, Bandung 40132, Indonesia;

[3] Dipartimento di Fisica, Università di Milano-Bicocca, Milan, 20126, Italy;

[4] CERN, Esplanade des Particules 1, 1211 Meyrin, Switzerland;

[5] Institute of Physics, Faculty of Physics, Astronomy, and Informatics, Nicolaus Copernicus University in Toruń, ul. Grudziądzka 5, 87-100 Toruń, Poland;

[6] School of Electrical and Electronic Engineering, Nanyang Technological University, Singapore 639798, Singapore;

[7] Institut Lumière Matière, UMR5306, Université Claude Bernard Lyon1 and CNRS Lyon, France;

[8] Dipartimento di Chimica Industriale "Toso Montanari", Università di Bologna, 40136 Bologna, Italy.

* Corresponding author:

Email addresses: daniele.cortecchia2@unibo.it (Daniele Cortecchia),

muhammad.birowosuto@port.lukasiewicz.gov.pl (Muhammad Danang Birowosuto)

Sheet List

Fig. S1. Rietveld refinement of crystal XRD diffractograms of (a) $(PEA)_2PbI_4$, (b) $(PEA)_2MAPb_2I_7$, (c) $(BA)_2PbI_4$, and (d) $(BA)_2MAPb_2I_7$ crystals. Reliability factors for $(PEA)_2PbI_4$: χ^2 = 5.25, R-factor = 5.79, R_p = 29.10, R_{wp} = 25.80. Reliability factors for $(PEA)_2MAPb_2I_7$: χ^2 = 3.22, R-factor = 2.46, R_p = 6.55, R_{wp} = 12.6. Reliability factors for $(BA)_2PbI_4$: χ^2 = 12.32, R-factor = 43.90, R_p = 15.90, R_{wp} = 12.30. Reliability factors for $(BA)_2MAPb_2I_7$: χ^2 = 3.40, R-factor = 13.50, R_p = 93.00, R_{wp} = 92.30. Insets correspond to the photographs of the crystal.

Fig. S2. Absorption spectra from (a) $(PEA)_2PbI_4$, (b) $(PEA)_2MAPb_2I_7$, (c) $(BA)_2PbI_4$, (d) $(BA)_2MAPb_2I_7$ and their fitting curves with Elliot method in Equations S1 and S2.

Fig. S3. (a) Absorption and PL spectra excited at 375 nm with a logarithmic scale of y-axis recorded at RT; (b) TRPL decay curve excited at 375 nm monitoring 420 nm emission at RT of (b) $(PEA)_2PbBr_4$, (c) $(BA)_2PbBr_4$ crystals. Insets correspond to the photographs of the crystals.

Fig. S4. TRPL decay curve excited at 532 nm monitoring 620 nm emission at RT of (a) $(PEA)_2PbI_4$, (b) $(PEA)_2MAPb_2I_7$, (c) $(BA)_2PbI_4$ crystals.

Fig. S5. RL spectra mapping at different temperatures from 10 to 350 K for (a) $(PEA)_2PbBr_4$, (b) $(BA)_2PbBr_4$, (c) $(PEA)_2PbI_4$, (d) $(PEA)_2MAPb_2I_7$, (e) $(BA)_2PbI_4$, and (f) $(BA)_2MAPb_2I_7$ crystals.

Fig. S6. Delay distributions at 10 mV with ^{22}Na source emitting two 511 keV γ-rays back-to-back of (a) $(BA)_2PbI_4$, (c) $(PEA)_2MAPb_2I_7$. The red solid lines are the Gaussian functions, which fit the distributions, and the black dotted lines provide the full width half maximum (FWHM) of the distributions. CTR_{sample} versus leading edge threshold values. CTR_{FWHM} fit to the data points is shown as blue dashed lines of (b) $(BA)_2PbI_4$, (d) $(PEA)_2MAPb_2I_7$.

Fig. S7. The normalized light yields calculated from pulse height spectra of $(PEA)_2MAPb_2I_7$ with exposing radiation time. A ^{137}Cs source, emitting 662 keV γ-rays, was used as an excitation and the sample was exposed to environmental conditions (standard atmosphere, 65% humidity).

1. X-ray diffraction, and crystal lattice parameters

The X-ray diffraction (XRD) spectrum in Figure S1 were analyzed the peaks and determined for the baseline. Then, using FullProf XRD software, we performed Rietveld refinement with the inputs from previous lattice parameters for $(PEA)_2PbI_4$,[1] $(PEA)_2MAPb_2I_7$,[2] $(BA)_2PbI_4$,[3] and $(BA)_2MAPb_2I_7$ crystals.[4] The refinement parameters were summarized in the manuscript Table I.

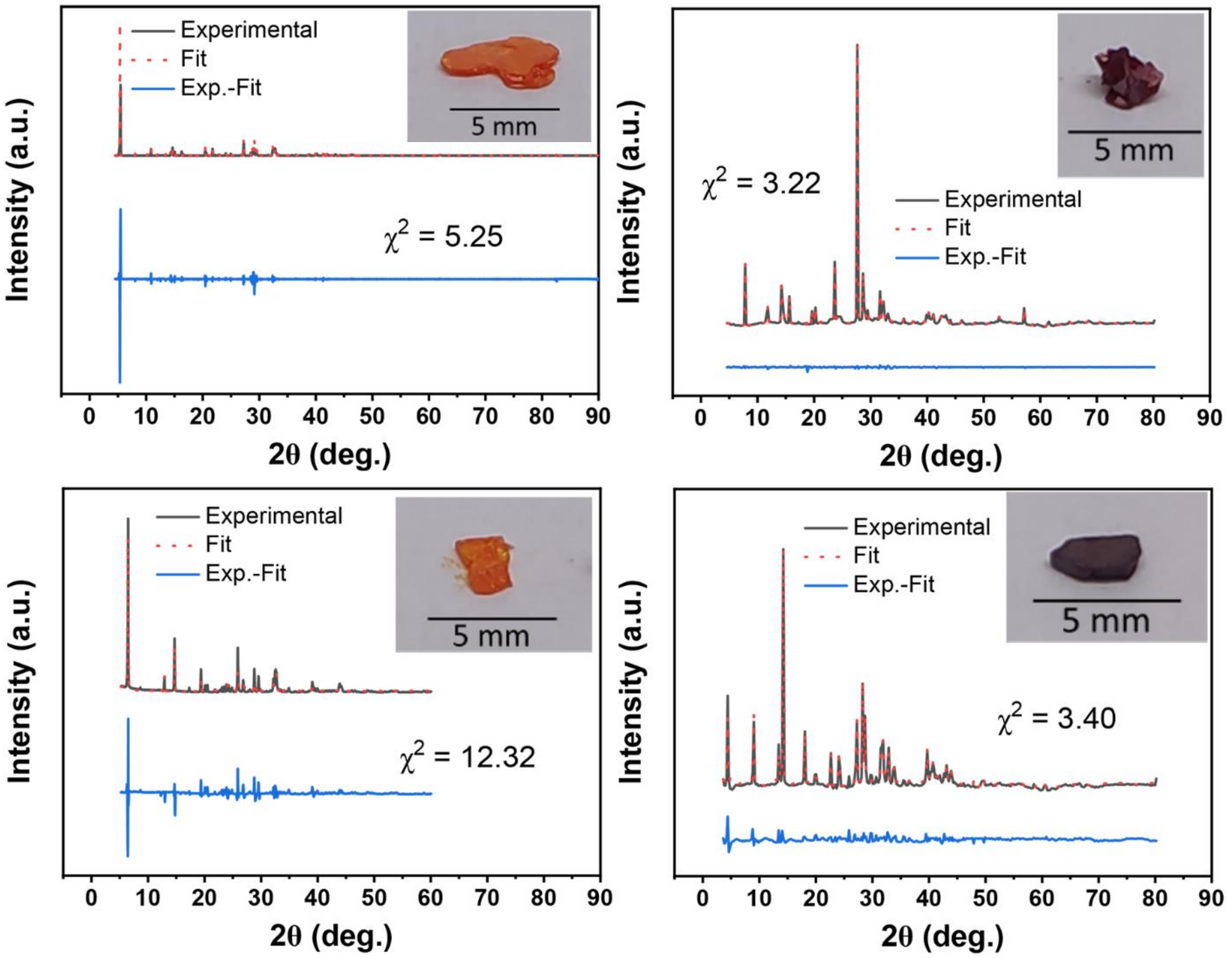

Fig. S1. Rietveld refinement of crystal XRD diffractograms of (a) $(PEA)_2PbI_4$, (b) $(PEA)_2MAPb_2I_7$, (c) $(BA)_2PbI_4$, and (d) $(BA)_2MAPb_2I_7$ crystals. Reliability factors for $(PEA)_2PbI_4$: χ^2 = 5.25, R-factor = 5.79, R_p = 29.10, R_{wp} = 25.80. Reliability factors for $(PEA)_2MAPb_2I_7$: χ^2 = 3.22, R-factor = 2.46, R_p = 6.55, R_{wp} = 12.6. Reliability factors for $(BA)_2PbI_4$: χ^2 = 12.32, R-factor = 43.90, R_p = 15.90, R_{wp} = 12.30. Reliability factors for $(BA)_2MAPb_2I_7$: χ^2 = 3.40, R-factor = 13.50, R_p = 93.00, R_{wp} = 92.30. Insets correspond to the photographs of the crystals.

2. Absorption spectra fitting

The fit to absorption spectrum in Fig. S2 was performed by Elliot formalism.[5] In principle, the contributions to the absorption coefficient (α) can be defined from free carriers (continuum) (α_c) and excitons (α_{ex}).

$$\alpha(\hbar\omega) = \alpha_c + \alpha_{ex} \qquad \text{(S1)}$$

$$\alpha(\hbar\omega) = P_{cv}\left[\,\theta(\hbar\omega - E_g)\cdot\left(\frac{\pi e^{\pi x}}{\sinh(\pi x)}\right) + R_{ex}\sum_{n=1}^{\infty}\frac{4\pi}{n^3}\cdot\delta\left(\hbar\omega - E_g + \frac{R_{ex}}{n^2}\right)\right] \qquad \text{(S2)}$$

where the frequency dependence of P_{cv} is a constant and related to the interband transition matrix element, $\hbar\omega$ is the photon energy, $\theta(\hbar\omega - E_g)$ is the heavyside step fuction, x is defined as p $R_{ex}/(\hbar\omega - E_g)$, and δ denotes a delta function, R_{ex} is exciton Rydberg energy, n is the principle quantum number. From the fits, obtained E_g^{abs} were summarized in manuscript Table I.

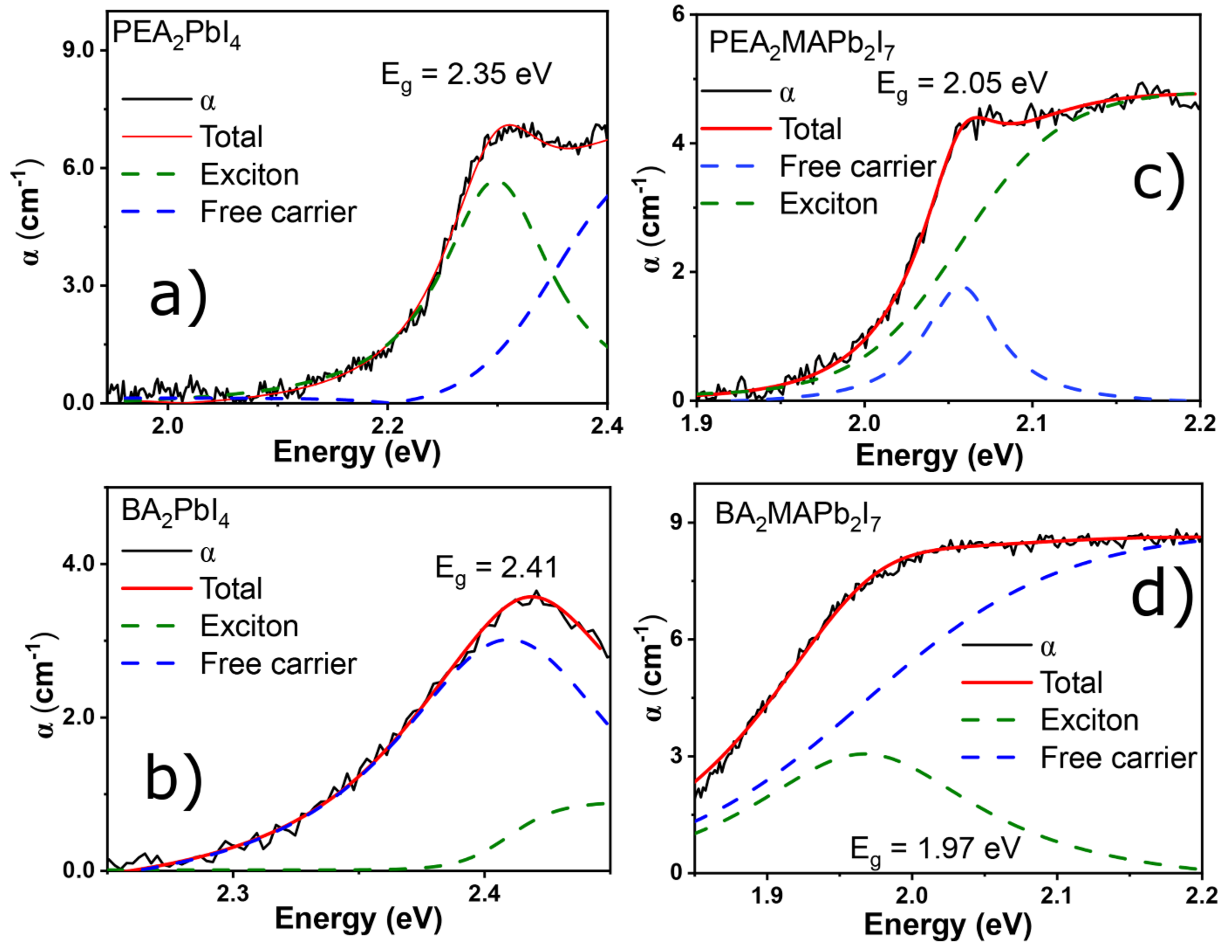

Fig. S2. Absorption spectra from (a) $(PEA)_2PbI_4$, (b) $(PEA)_2MAPb_2I_7$, (c) $(BA)_2PbI_4$, (d) $(BA)_2MAPb_2I_7$ and their fitting curves with Elliot method in Equations S1 and S2.

3. Absorption, PL and TRPL decay

Absorption and PL spectra excited at 375 nm of $(PEA)_2PbBr_4$ and $(BA)_2PbBr_4$ crystals at RT are shown in Fig. S3(a). Absorption peaks at 401 nm, 346 nm and calculated band gap from Elliot fit of 3.09 eV and 3.59 eV shown in Fig. S2(a, b) were observed for $(PEA)_2PbBr_4$ and $(BA)_2PbBr_4$ crystals, respectively. PL peaks excited at 375 nm at RT exhibit 438 nm (2.83 nm) and 434 nm (2.86 eV) for $(PEA)_2PbBr_4$ and $(BA)_2PbBr_4$ crystals, respectively. The TRPL spectra were well fitted by tri-exponential decays, as shown in Fig. S3(b, c) for $(PEA)_2PbBr_4$ and $(BA)_2PbBr_4$ crystals. The decay components for $(PEA)_2PbBr_4$ crystal of 0.1 ns, 2.9 ns and 8.3 ns correspond to the exciton emission as the decay curve was recorded by monitoring 420 nm emission wavelength and an average decay times τ_{avg}^{PL} of 5.9 ns.[6] On the other hand, the decay components for $(BA)_2PbBr_4$ crystal of 0.8 ns, 2.5 ns and 8.8 ns correspond to the exciton emission as the decay curve was recorded by monitoring 420 nm emission wavelength and the average value of decay times τ_{avg}^{PL} of 3.3 ns.[6]

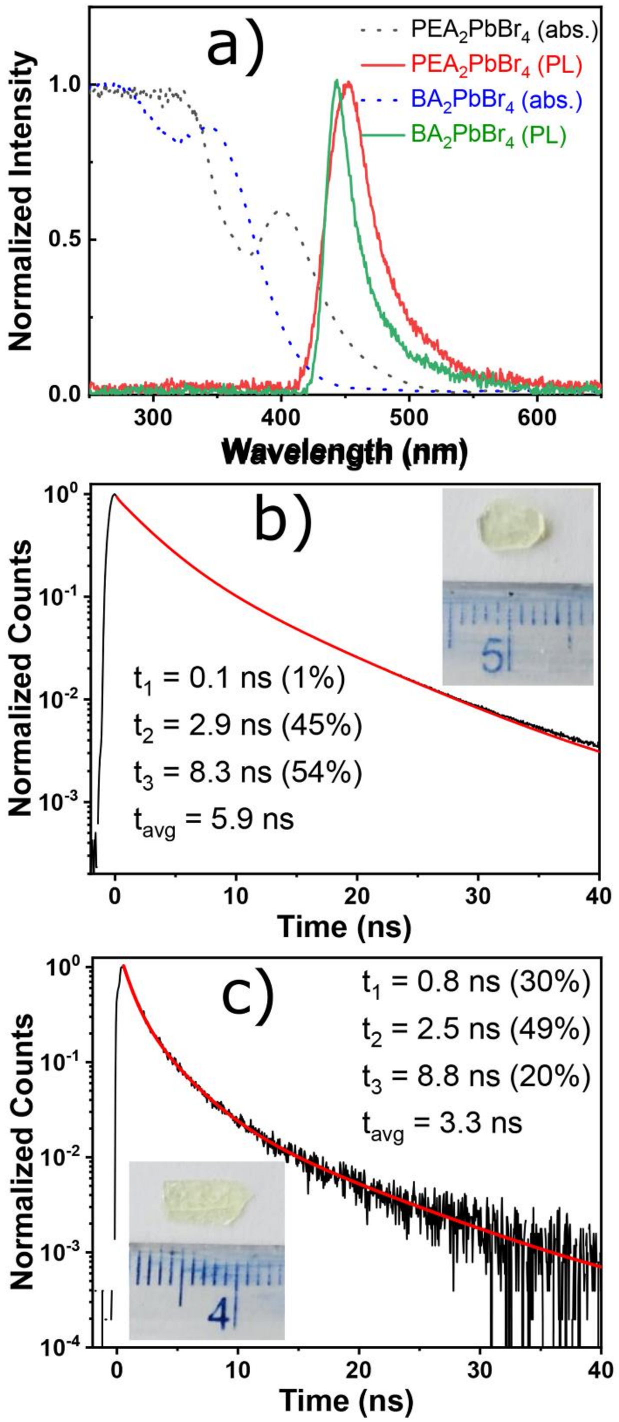

Fig. S3. (a) Absorption and PL spectra excited at 375 nm with a logarithmic scale of y-axis recorded at RT; (b) TRPL decay curve excited at 375 nm monitoring 420 nm emission at RT of (b) $(PEA)_2PbBr_4$, (c) $(BA)_2PbBr_4$ crystals. Insets correspond to the photograph of the crystals.

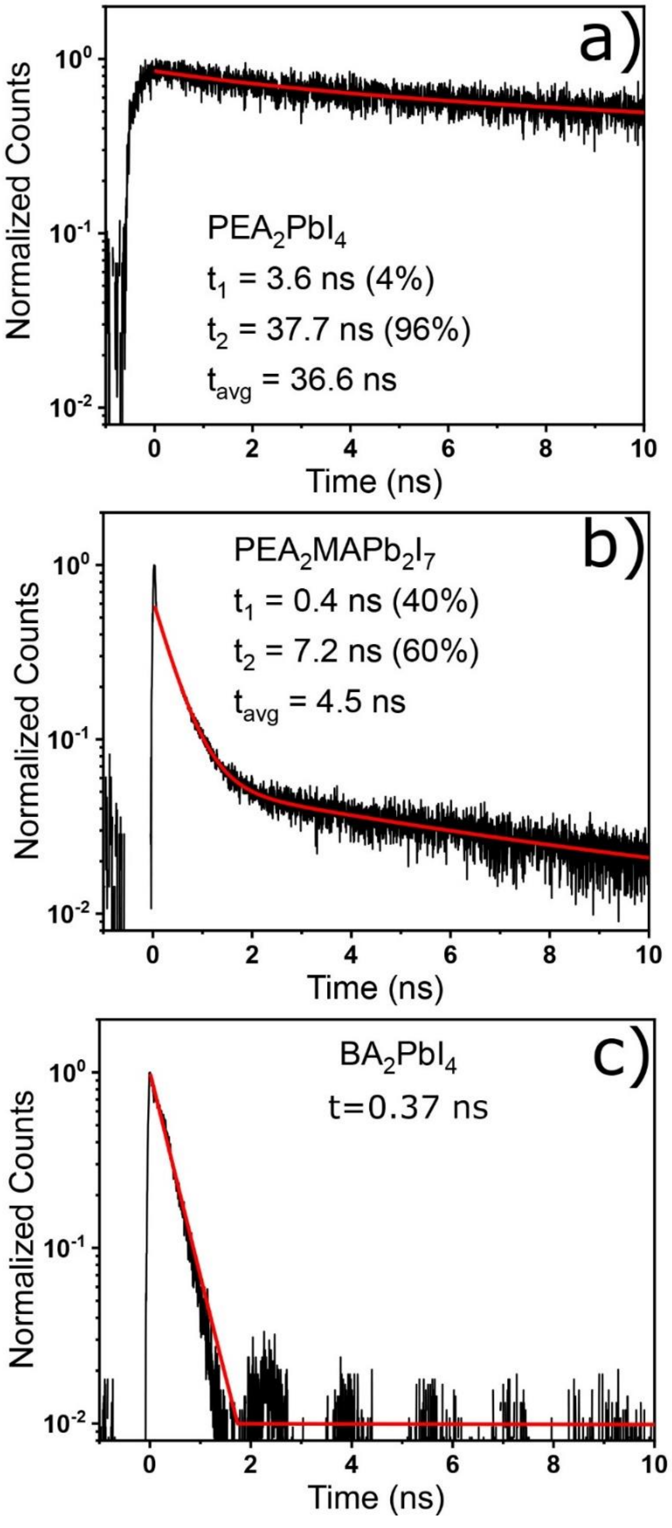

Fig. S4. TRPL decay curve excited at 532 nm monitoring 620 nm emission at RT of (a) $(PEA)_2PbI_4$, (b) $(PEA)_2MAPb_2I_7$, (c) $(BA)_2PbI_4$ crystals.

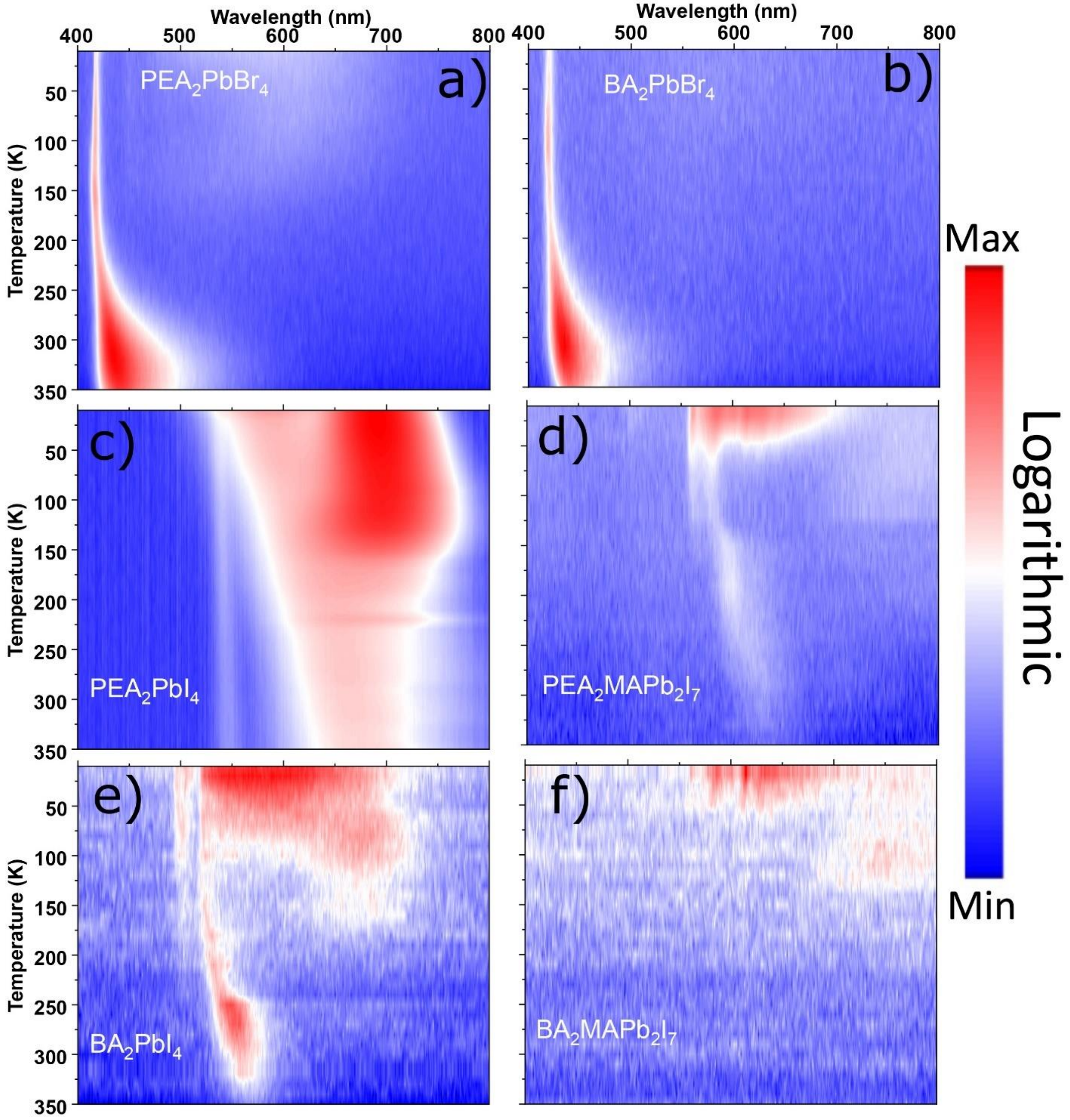

Fig. S5. RL spectra mapping at different temperatures from 10 to 350 K for (a) $(PEA)_2PbBr_4$, (b) $(BA)_2PbBr_4$, (c) $(PEA)_2PbI_4$, (d) $(PEA)_2MAPb_2I_7$, (e) $(BA)_2PbI_4$, and (f) $(BA)_2MAPb_2I_7$ crystals.

4. Delay distribution and CTR measurements

The CTR measurement results are shown in Fig. S6 (a, b) and (c, d) for $(BA)_2PbI_4$ and $(PEA)_2MAPb_2I_7$ crystals, respectively. The details of the analysis and the experiments were similar to those previously reported for $(PEA)_2PbBr_4$ crystal,[7] except the optical coupling which was not applied to the crystals due to samples hygroscopicity. Since we used LSO:Ce:Ca scintillator as a reference, the measured CTR (CTR_{meas}) is given by a combination of the CTR of the crystal and that of the reference.[8] In order to evaluate the CTR of two identical samples (CTR_{sample}), the contribution of the reference crystal (CTR_{ref}) was subtracted in quadrature from the measured CTR (CTR_{meas}) as shown in the following equation:

$$CTR_{sample} = \sqrt{2 \cdot CTR_{meas}{}^{2} - CTR_{ref.}{}^{2}} \qquad \text{(S3)}$$

From Fig. S6(a), (c), we obtained CTR_{meas} from the full width half maximum (FWHM) of the delay distributions of the events with 511 keV energy depositions in both detectors. After subtracting CTR_{ref} of 61 ps via Eq. S3, we obtained CTR_{sample} of 149±10 ps, 207±14 ps for $(BA)_2PbI_4$ and $(PEA)_2MAPb_2I_7$ crystals, respectively as summarized in Table S4. To determine the best CTR value, a series of CTR measurements were performed varying the leading-edge threshold set on the oscilloscope. Fig. S6(b), (d) exhibits CTR_{sample} values versus leading edge threshold values. Since there is an effect of limiting the electronic bandwidth and an influence of the electronic noise to CTR_{FWHM}, [9,10] the data points were fitted by using an empirical model formulated in[11,12]:

$$CTR_{FWHM}(v|p_0, p_1, p_2) = \sqrt{p_0{}^2 + (p_1 . v)^2 + \left(\frac{p_2}{v}\right)^2} \qquad \text{(S4)}$$

where v, p_0, p_1, p_2 are the leading-edge threshold, the intrinsic limit of CTR, the photostatistics effect, and the noise contributions, respectively. From the fits in Fig. S6(b) and (d), the minimum value CTR_{min} of CTR_{FWHM} for $(BA)_2PbI_4$ and $(PEA)_2MAPb_2I_7$ crystals were determined to be 149 ps and 207 ps, respectively. Both sample CTR are worse than 81 ps of $(BA)_2PbBr_4$ crystal.[13,14]

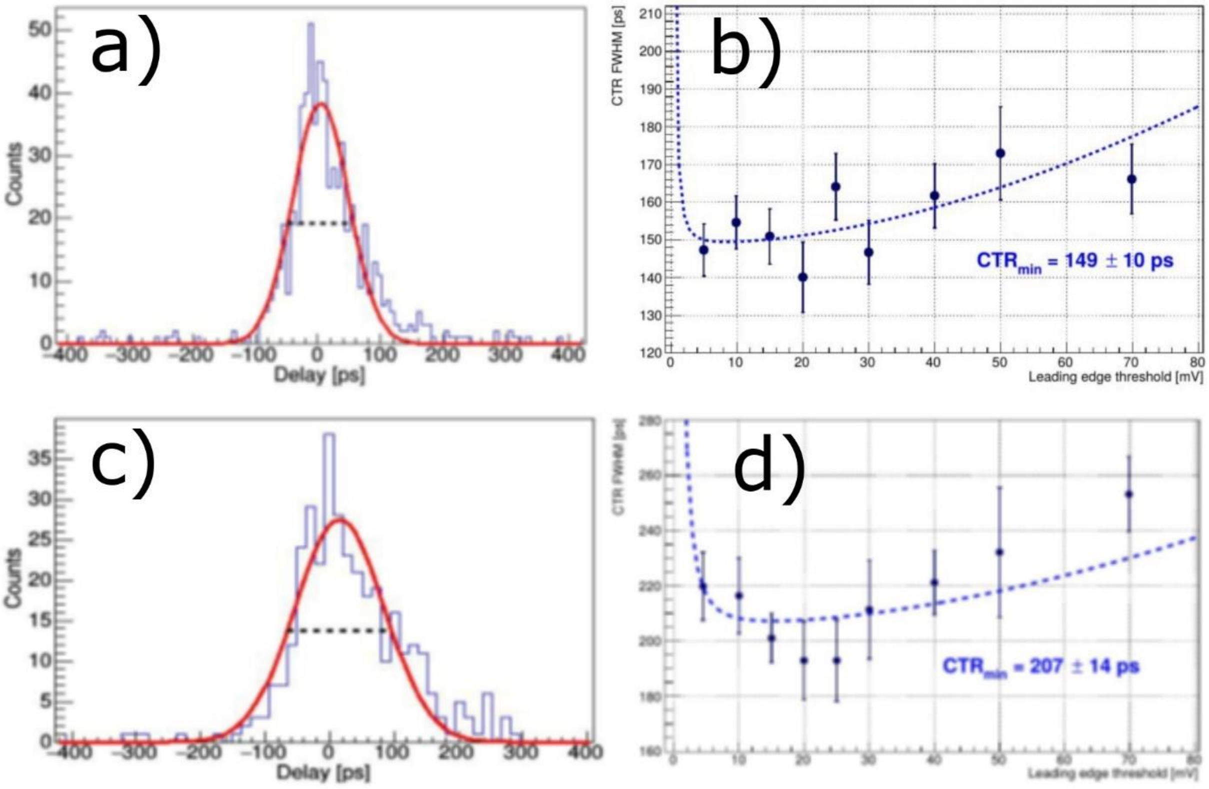

Fig. S6. Delay distributions at 10 mV with ^{22}Na source emitting two 511 keV γ-rays back-to-back of (a) $(BA)_2PbI_4$, (c) $(PEA)_2MAPb_2I_7$. The red solid lines are the Gaussian functions, which fit the distributions, and the black dotted lines provide the full width half maximum (FWHM) of the distributions. CTR_{sample} versus leading edge threshold values. CTR_{FWHM} fit to the data points is shown as blue dashed lines of (b) $(BA)_2PbI_4$, (d) $(PEA)_2MAPb_2I_7$.

5. Light yield stability as a function of time

We measured the pulse height spectra of the $(PEA)_2MAPb_2I_7$ sample for 6 hours and the derived values of the light yield were plotted with the normalized values at the initial time as shown in **Fig. S7**. To test the stability of the light yield of $(PEA)_2MAPb_2I_7$, the ambient environmental conditions with standard atmosphere and 65% humidity were recorded.

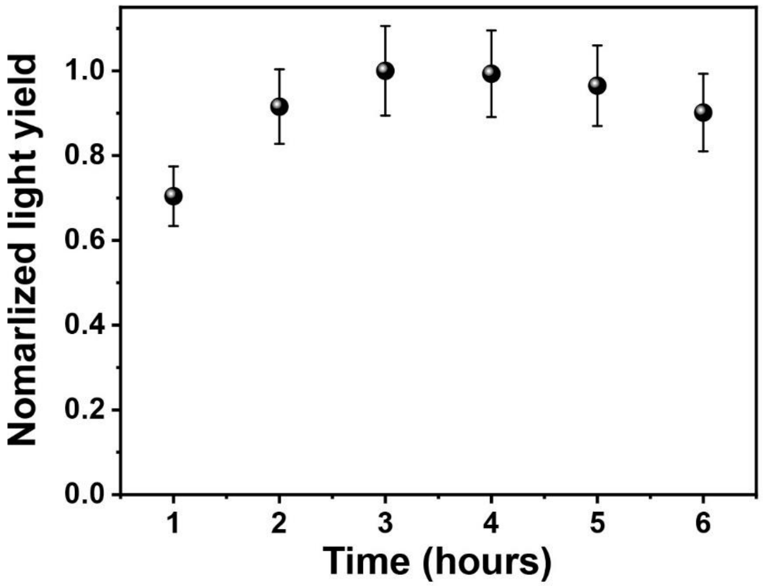

Fig. S7. The normalized light yields calculated from pulse height spectra of $(PEA)_2MAPb_2I_7$ with exposing radiation time. A ^{137}Cs source, emitting 662 keV γ-rays, was used as an excitation and the sample was exposed to environmental conditions (standard atmosphere, 65% humidity).

Table S1 Afterglow decay parameters

	Comp. 1 (s/%)	Comp. 2 (s/%)	Comp. 3 (s/%)	Average decay time (s)
$(PEA)_2PbI_4$	4.9/10	33.1/32	364.3/58	224.1
$(PEA)_2MAPb_2I_7$	9.6/1	121.6/4	4560.1/95	4336.5
$(BA)_2PbI_4$	0.2/2	15.7/26	166.5/72	87.4
$(BA)_2MAPb_2I_7$	6.5/35	66.5/65	-	45.4

Table S2 Thermoluminescence parameters

	T_{max} (K)	E (meV)	N_0 (a.u.)	σ (s^{-1})
$(PEA)_2PbI_4$	47<br>101<br>144	26<br>85<br>199	$1.2x10^4$<br>$1.3x10^4$<br>$2.1x10^3$	$1.5x10^3$<br>$3.2x10^3$<br>$1.2x10^3$
$(PEA)_2MAPb_2I_7$	35<br>46<br>88<br>150<br>200	86.1<br>121.9<br>200.6<br>337.3<br>492.8	$1.8x10^4$<br>$3.0x10^4$<br>$6.6x10^4$<br>$2.2x10^5$<br>$1.7x10^5$	$7.3x10^3$<br>$3.5x10^3$<br>$1.5x10^3$<br>$8.9x10^2$<br>$8.9x10^2$
$(BA)_2PbI_4$	54<br>127	31<br>146	$2.9x10^3$<br>$1.5x10^4$	$1.9x10^5$<br>$3.8x10^3$
$(BA)_2MAPb_2I_7$	39<br>78	21<br>32	$1.1x10^4$<br>$5.5x10^3$	$1.4x10^5$<br>$4.2x10^3$

Table S3 Summary of average time-resolved photoluminescent (TRPL), light yield at RT and at 10 K for $(PEA)_2PbI_4$, $(PEA)_2MAPbI_4$, $(PEA)_2PbBr_4$, $(BA)_2PbI_4$, $(BA)_2MAPbI_4$, $(BA)_2PbBr_4$ crystals.

	Avg. TRPL decay time (ns)	Light yield @ RT (photons/keV)	Light yield @10 K (photons/keV)	Ref. No.
$(PEA)_2PbI_4$	1.0	1	10	This work
$(PEA)_2MAPbI_4$	0.9	1.4	8.4	This work
$(PEA)_2PbBr_4$	5.9	10	7	This work and [15]
$(BA)_2PbI_4$	0.24	2	10	This work
$(BA)_2MAPbI_4$	0.5	<0.6	7.5	This work
$(BA)_2PbI_4$	0.5	0.1	0.6	[16]
$(BA)_2PbBr_4$	3.3	40	22	This work and [17]

Table S4 Gamma-ray excited scintillation decay parameters

	Comp. 1 (ns/%)	Comp. 2 (ns/%)	Comp. 3 (ns/%)	Average decay time (ns)	CTR (ps)
$(PEA)_2PbI_4$	0.5/10	22/48	450/40	190	138±10
$(PEA)_2MAPb_2I_7$	9.3/37	43/23	249/39	112	207±14
$(BA)_2PbI_4$	0.4/2	15/72	380/26	111	149±10
$(BA)_2MAPb_2I_7$	-	-	-	-	-

REFERENCES

(1) K. -Z. Du; Q. Tu; X. Zhang; Q. Han; J. Liu; S. Zauscher; Mitzi, D. B. Two-dimensional lead(ii) halide-based hybrid perovskites templated by acene alkylamines: crystal structures, optical properties, and piezoelectricity. *Inorg. Chem.* **2017**, *56*, 9291-9302. DOI: 10.1021/acs.inorgchem.7b01094.

(2) J. Song; Y. Dang; X . L. Liua; Tao, X. Layered hybrid lead perovskite single crystals: phase transformations and tunable optical properties. *CrystEngComm* **2020**, *22*, 6310-6315. DOI: 10.1039/d0ce00753f.

(3) Mitzi, D. B. Synthesis, crystal structure, and optical and thermal properties of $(C_4H_9NH_3)_2MI_4$ (M = Ge, Sn, Pb). *Chem. Mater.* **1996**, *8* (3), 791-800. DOI: 10.1021/cm9505097.

(4) J. Yin; R. Naphade; L. G. Arzaluz; J. -L. Brédas; O. M. Bakr; Mohammed, O. M. Modulation of broadband emissions in two dimensional ⟨100⟩-oriented ruddlesden-popper hybrid perovskites. *ACS Energy Lett.* **2020**, *5*, 2149-2155. DOI: 10.1021/acsenergylett.0c01047.

(5) Elliott, R. J. Theory of the effect of spin-orbit coupling on magnetic resonance in some semiconductors. *Phys. Rev.* **1954**, *96* (2), 266-279. DOI: 10.1103/PhysRev.96.266.

(6) F. Maddalena; M. H. Mahyuddin; D. Kowal; M. E. Witkowski; M. Makowski; Md A. K. Sheikh; S. Mahato; R. Je̜drzejewski; W. Drozdowski; C. Dujardin; et al. Lattice expansion in rubidium doped hybrid organic-inorganic perovskite crystals resulting smaller-bandgap and higher-light-yield scintillators. *Inorg. Chem.* **2023 (Under review)**.
(7) Cala', R.; Frank, I.; Pagano, F.; Maddalena, F.; Dang, C.; Birowosuto, M. D.; Auffray, E. Sub-100-picosecond time resolution from undoped and Li-doped two-dimensional perovskite scintillators. *Appl. Phys. Lett.* **2022**, *120* (24), 241901. DOI: 10.1063/5.0093606.
(8) E. Auffray; B. Frisch; F. Geraci; A. Ghezzi; S. Gundacker; H. Hillemanns; P. Jarron; T. Meyer; M. Paganoni; K. Pauwels; et al. A comprehensive and systematic study of coincidence time resolution and light yield using scintillators of different size, wrapping and doping. *IEEE Trans. Nucl. Sci.* **2013**, *60* (5), 3163-3171. DOI: 10.1109/NSSMIC.2011.6154402.
(9) J. W. Cates, S. G., E. Auffray, P. Lecoq, C. S. Levin Improved single photon time resolution for analog SiPMs with front end readout that reduces influence of electronic noise. *Phys. Med. Biol.* **2018**, *63* (18), 185022. DOI: 10.1088/1361-6560/aadbcd.
(10) S. Gundacker, R. M. T., E. Auffray, M. Paganoni, P. Lecoq. High frequency SiPM readout advances measured coincidence time resolu tion limits in TOF-PET. *Phys. Med. Biol.* **2019**, *64* (5), 055012. DOI: 10.1088/1361-6560/aafd52.
(11) S. Gundacker; E. Auffray; B. Frisch; P. Jarron; A. Knapitsch; T. Meyer; M. Pizzichemi; Lecoq, P. Time of flight positron emission tomography towards 100 ps resolution with L(Y)SO: an experimental and theoretical analysis. *J. Instrum.* **2013**, *8* (07), P07014–P07014. DOI: 10.1088/1748-0221/8/07/P07014.
(12) S. Gundacker; R. M. Turtos; N. Kratochwil; R. H. Pots; M. Paganoni; P. Lecoq; Auffray, E. Experimental time resolution limits of modern SiPMs and TOF-PET detectors exploring different scintillators and cherenkov emission. *Phys. Med. Biol.* **2020**, *65* (2), 025001. DOI: 10.1088/1361-6560/ab63b4.
(13) F. Maddalena; A. Xie; Arramel; M. E. Witkowski; M. Makowski; B. Mahler; W. Drozdowski; T. Mariyappan; S. V. Springham; P. Coquet; et al. Effect of commensurate lithium doping on the scintillation of two-dimensional perovskite crystals. *J. Mater. Chem. C* **2021**, *9*, 2504-2512. DOI: 10.1039/d0tc05647b.
(14) Md A. K. Sheikh; D. Kowal; M. H. Mahyuddin; D. Onggo; F. Maddalena; C. Dang; R. Cala'; E. Auffray; M. E. Witkowski; M. Makowski; et al. Solution-processable A_2XY_4 (A=PEA, BA; X= Pb, Sn, Cu, Mn; Y=Cl, Br, I) crystals for high light yield and ultrafast scintillators. *IEEE Trans. Nucl. Sci.* **2023 (Accepted)**. DOI: 10.1109/TNS.2023.3267636.
(15) Xie, A.; Hettiarachchi, C.; Maddalena, F.; Witkowski, M. E.; Makowski, M.; Drozdowski, W.; Arramel, A.; Wee, A. T. S.; Springham, S. V.; Vuong, P. Q.; et al. Lithium-doped two-dimensional perovskite scintillator for wide-range radiation detection. *Commun. Mater.* **2020**, *1* (1), 37. DOI: 10.1038/s43246-020-0038-x.
(16) A. Xie; F. Maddalena; M. E. Witkowski; M. Makowski; B. Mahler; W. Drozdowski; S. V. Springham; P. Coquet; C. Dujardin; M. D. Birowosuto; et al. Library of two-dimensional hybrid lead halide perovskite scintillator crystals. *Chem. Mater.* **2020**, *32* (19), 8530-8539. DOI: 10.1021/acs.chemmater.0c02789.
(17) Diguna, L. J.; Jonathan, L.; Mahyuddin, M. H.; Arramel; Maddalena, F.; Mulyani, I.; Onggo, D.; Bachiri, A.; Witkowski, M. E.; Makowski, M.; et al. BA_2XBr_4 (X = Pb, Cu, Sn): from lead to lead-free halide perovskite scintillators. *Mater. Adv.* **2022**, *3* (12), 5087-5095, 10.1039/D2MA00258B. DOI: 10.1039/D2MA00258B.
